# MIRit: an integrative R framework for the identification of impaired miRNA–mRNA regulatory networks in complex diseases

**DOI:** 10.1093/bioadv/vbag042

**Published:** 2026-02-13

**Authors:** Jacopo Ronchi, Maria Foti

**Affiliations:** School of Medicine and Surgery, University of Milano-Bicocca, Monza (MB), 20900, Italy; PhD Program in Neuroscience, School of Medicine and Surgery, University of Milano-Bicocca, Monza (MB), 20900, Italy; School of Medicine and Surgery, University of Milano-Bicocca, Monza (MB), 20900, Italy; BicOMICs, University of Milano-Bicocca, Monza (MB), 20900, Italy

## Abstract

**Motivation:**

MicroRNAs (miRNAs) play a central role in controlling gene expression, and their abnormal activity is frequently linked to disease. Despite advancements in transcriptomic technologies, elucidating miRNA-mediated mechanisms remains challenging due to methodological limitations and a lack of standardized frameworks.

**Results:**

To overcome these barriers, we developed MIRit, a comprehensive R package designed for the rigorous analysis of miRNA–mRNA interactions. With flexible support for both matched and unmatched datasets, MIRit leverages cutting-edge target identification strategies and applies suitable statistical approaches for each scenario. In this study, we benchmarked the performance of commonly used statistical tests for integrative miRNA analysis and demonstrated the effectiveness of MIRit across three human disease contexts—dilated cardiomyopathy, clear cell renal cell carcinoma, and Alzheimer’s disease—by uncovering functionally relevant miRNA–target disruptions consistent with known disease mechanisms. Through its streamlined pipeline and biologically appropriate methods, MIRit enables more reproducible and accurate insights into the complex landscape of post-transcriptional regulation.

**Availability and implementation:**

The tool is fully open-source and freely accessible via Bioconductor (https://bioconductor.org/packages/release/bioc/html/MIRit.html), making it readily available to the broader scientific community.

## 1 Introduction

MicroRNAs (miRNAs) are a class of small non-coding RNAs with an average length of 22 nucleotides, whose role is mainly to negatively regulate gene expression at a post-transcriptional stage. After their biogenesis, they typically promote mRNA degradation and translation inhibition by interacting with the 3’ untranslated region (3’ UTR) of target genes (O’Brien et al. 2018). Over the last few decades, evidence has progressively shown the involvement of miRNAs in mammalian pathways. Currently, it is estimated that more than 60% of human genes are controlled by miRNAs (Friedman *et al.* 2009). Given their extensive regulatory activity, unbalanced miRNA expression can have dramatic consequences on cellular activities, and their dysregulation has been associated with several diseases (Ardekani and Naeini 2010). In light of this, there has been a growing interest in the study of miRNAs with the aim of characterizing the regulatory networks involved in human disorders. Several approaches exist to quantify miRNA abundance at the omic level, including microarrays and miRNA-Seq. Consequently, the determination of differentially expressed miRNAs (DE-miRNAs) in different biological conditions can be successfully performed using the same analytical procedures commonly employed for transcriptomics. However, even after their quantification, elucidating the biological consequences of dysregulated miRNAs is highly challenging. The limited functional annotation of miRNAs hinders our understanding of miRNA-driven mechanisms in complex diseases, likely due to the ambiguity of miRNA–target gene interactions across cell types and conditions. One approach to addressing this issue is to combine miRNA and gene expression levels to evaluate the impact of each miRNA on the expression of its targets, thereby reconstructing perturbed molecular networks.

Despite the advantages of integrative analyses, however, current approaches are rarely successful or reproducible. In particular, miRNA–mRNA analyses are dramatically influenced by the choice of miRNA target genes. In this regard, the use of different prediction algorithms can drastically affect the results of a study, thus limiting the reproducibility of the conclusions drawn. Furthermore, the statistical methods used to analyze the relationship between miRNA and gene expression are often inaccurate and unreliable. The most widely used approach at present is correlation analysis, which quantifies the influence of miRNAs on the expression of their target genes. However, incorrect implementation often reduces the statistical power to detect meaningful interactions. Moreover, relying solely on correlation approaches severely limits the availability of datasets for integrative analyses, as these methods require matched miRNA and mRNA expression measurements from the same specimens.

In this regard, existing R/Bioconductor packages primarily focus on the integrative analysis of paired miRNA–mRNA expression data. While these tools—such as miRLAB (Le *et al.* 2015), MiRComb (Vila-Casadesús *et al.* 2016), anamiR (Wang *et al.* 2019), mirTarRnaSeq (Movassagh *et al.* 2022), SpidermiR (Cava *et al.* 2017), and TimiRGeN (Patel *et al.* 2021)—implement diverse methods for analyzing regulatory interactions, they do not currently support the analysis of unpaired datasets.

Besides, almost all approaches suffer from common drawbacks. Firstly, most methods use a combination of multiple databases to define miRNA targets, which often results in an unreasonably high number of interactions and an inflated rate of false positive hits. This complicates subsequent analysis by increasing the burden of multiple testing and limiting interpretation and experimental validation. Secondly, some of the implemented statistical approaches are not always appropriate for miRNA and mRNA datasets. For instance, lasso and elastic-net regularized models should not be used when the number of miRNAs targeting a gene exceeds the number of samples, which is often the case for some central hub genes.

To address all these issues and fill these gaps, we developed MIRit, an open-source, comprehensive and all-in-one R framework that encompasses all the steps required to perform an integrative miRNA–mRNA analysis. Unlike other tools, MIRit is designed to work with data from multiple technologies, and implements statistical approaches that allow the analysis of unpaired datasets. In this study, we present the implementation of MIRit, compare the performance of widely used methods for miRNA–mRNA integration on synthetic datasets, and demonstrate the utility of MIRit in the context of human disease using both paired and unpaired datasets.

## 2 Methods

### 2.1 Software’s architecture

MIRit is an integrative R framework designed to uncover dysfunctional miRNA–mRNA regulatory networks in complex diseases by combining expression profiling, functional annotation, and statistical integration. The pipeline provides a complete and modular workflow, compatible with both paired and unpaired datasets, enabling flexible analyses across diverse transcriptomic platforms.

The workflow begins with differential expression analysis of miRNAs and mRNAs using established R packages such as limma (Ritchie *et al.* 2015), edgeR (Robinson *et al.* 2010), or DESeq2 (Love *et al.* 2014), depending on whether the input data are derived from microarrays or RNA sequencing. Users can fully customize model parameters or import external results, allowing integration of measurements from other omics modalities. MIRit then performs functional enrichment of dysregulated genes using over-representation analysis (ORA) (Boyle *et al.* 2004), gene set enrichment analysis (GSEA) (Subramanian *et al.* 2005), or CAMERA (Wu and Smyth 2012), supporting major pathway repositories such as Gene Ontology (GO) (Aleksander et al. 2023), KEGG (Kanehisa and Goto 2000), Reactome (Gillespie *et al.* 2022), WikiPathways (Martens *et al.* 2021), and MSigDB (Subramanian *et al.* 2005). Next, MIRit examines potential genetic determinants of miRNA dysregulation by mapping DE-miRNAs to disease-associated variants obtained from the NHGRI-EBI GWAS Catalog (Sollis *et al.* 2023) through the gwasrapidd package (Magno and Maia 2020). The tool then retrieves miRNA targets from both experimentally validated interactions in miRTarBase (Cui *et al.* 2025) and predicted interactions aggregated in mirDIP (Hauschild *et al.* 2023), which combines results from 24 prediction algorithms using a unified scoring framework. Nevertheless, MIRit is also compatible with custom user-provided interactions, thereby allowing the use of miRNA–target interactions defined according to any method of choice. Finally, MIRit integrates miRNA and mRNA dysregulations to identify anti-correlated pairs. For paired datasets, it employs non-parametric correlation analyses, while for unpaired datasets, it tests directional associations using Fisher’s or Boschloo’s exact tests—with optional Lancaster’s mid-*P* correction—or the fry rotation gene set approach (Wu *et al.* 2010).

By seamlessly combining these analyses, MIRit offers a flexible and statistically rigorous platform for reconstructing disease-specific miRNA–mRNA interaction networks. A comprehensive description of the algorithmic steps and underlying statistical approaches is provided in Supplementary Text 1.1–1.5, available as supplementary data at *Bioinformatics Advances* online.

### 2.2 Dilated cardiomyopathy (DCM) dataset

As a first demonstration of MIRit’s usage in integrative miRNA–mRNA analysis, we examined myocardial biopsies from patients with DCM to reveal etiology-specific miRNA–mRNA interactions. In particular, we used a paired dataset that is publicly available on the Gene Expression Omnibus (GEO) database (Clough and Barrett 2016) under the accession number GSE243406 (Bonet *et al.* 2024). This dataset includes miRNA and mRNA expression measurements from four individuals with volume overload DCM (VCM) and four individuals with ischemic cardiomyopathy (ICM). Briefly, differential expression analysis of both miRNAs and mRNAs was performed using DESeq2. Features with a Storey’s *q*-value <0.05 were considered significant. GSEA was conducted on GO biological processes, applying a Benjamini-Hochberg adjusted *P*-value <0.1 as the significance threshold. To identify influential miRNA–target pairs, Spearman’s correlation was calculated, retaining pairs with ρ<−0.5 and adjusted *P*-value <0.1. A detailed description of the analytical procedures is provided in Supplementary Text 2, available as supplementary data at *Bioinformatics Advances* online.

### 2.3 Clear cell renal cell carcinoma (ccRCC) dataset

The performance of MIRit was also evaluated using a paired miRNA–mRNA dataset involving individuals diagnosed with ccRCC. This dataset is available on GEO (accession number GSE16441) and comprises miRNA and mRNA expression data from 17 ccRCC samples and 17 adjacent healthy tissues from the same donors (Liu *et al.* 2010). Raw microarray intensities were background-corrected and quantile-normalized. Lowly expressed probes were filtered, and differential expression analysis of both miRNAs and mRNAs was carried out using limma. Patient identity was included as a covariate to account for within-donor correlations. Features with absolute fold-change >1.5 and Benjamini-Hochberg adjusted *P*-value <0.05 were considered significant. Spearman’s correlation was then applied to identify anti-correlated miRNA–target pairs (ρ<−0.5 and adjusted *P*-value <0.1). Finally, ORA of affected target genes was performed using GO biological processes categories, with significance set at adjusted *P*-value <0.1. Further elucidation regarding pre-processing and analysis of this dataset can be found in Supplementary Text 3, available as supplementary data at *Bioinformatics Advances* online.

### 2.4 Alzheimer’s disease (AD) dataset

In this case study, two datasets involving different donors were used to demonstrate MIRit’s usage in cases where sample correspondence is missing. Specifically, miRNA expression in the Brodmann area 9 (BA9) of AD patients was evaluated using a small RNA-Seq experiment publicly available on GEO (accession GSE63501) (Santa-Maria *et al.* 2015). Meanwhile, mRNA expression in the same Brodmann area was assessed using a microarray experiment by Low *et al.* (2021), which is also publicly available on GEO (accession: GSE150696). For the miRNA dataset, FASTQ files were trimmed, quality-checked, and aligned using the miRge 3.0 pipeline (Patil and Halushka 2021). DE-miRNAs were then identified with limma-voom (Law *et al.* 2014), applying Storey’s *q*-value <0.1. For the mRNA dataset, raw microarray intensities were normalized with the robust multi-array average (RMA) algorithm as implemented in the oligo package (Carvalho and Irizarry 2010). Lowly expressed genes were discarded, and DEGs were identified using limma (adjusted *P*-value <0.05). Boschloo’s test was applied to detect miRNAs affecting gene expression (adjusted *P*-value <0.1). Finally, ORA of targets of influential miRNAs was performed using GO biological processes categories (adjusted *P*-value <0.1). Further details on the preliminary processing and examination of these datasets is provided in Supplementary Text 4, available as supplementary data at *Bioinformatics Advances* online.

### 2.5 Multiple testing correction

To control the false discovery rate (FDR) across multiple comparisons, we employed either the Storey’s *q*-value procedure (Storey 2002) or the Benjamini-Hochberg (BH) correction, depending on the characteristics of the data. Storey’s method was applied when the distribution of *P*-values under the null hypothesis was approximately uniform, as this approach estimates the proportion of true null hypotheses ($\pi_{0}$) and typically provides greater statistical power. When this assumption was not satisfied—indicating potential deviations from uniformity or dependency structures that could bias $\pi_{0}$ estimation—we instead used the more conservative BH correction, which maintains valid FDR control under broader conditions.

## 3 Results and discussion

**Figure 1:**
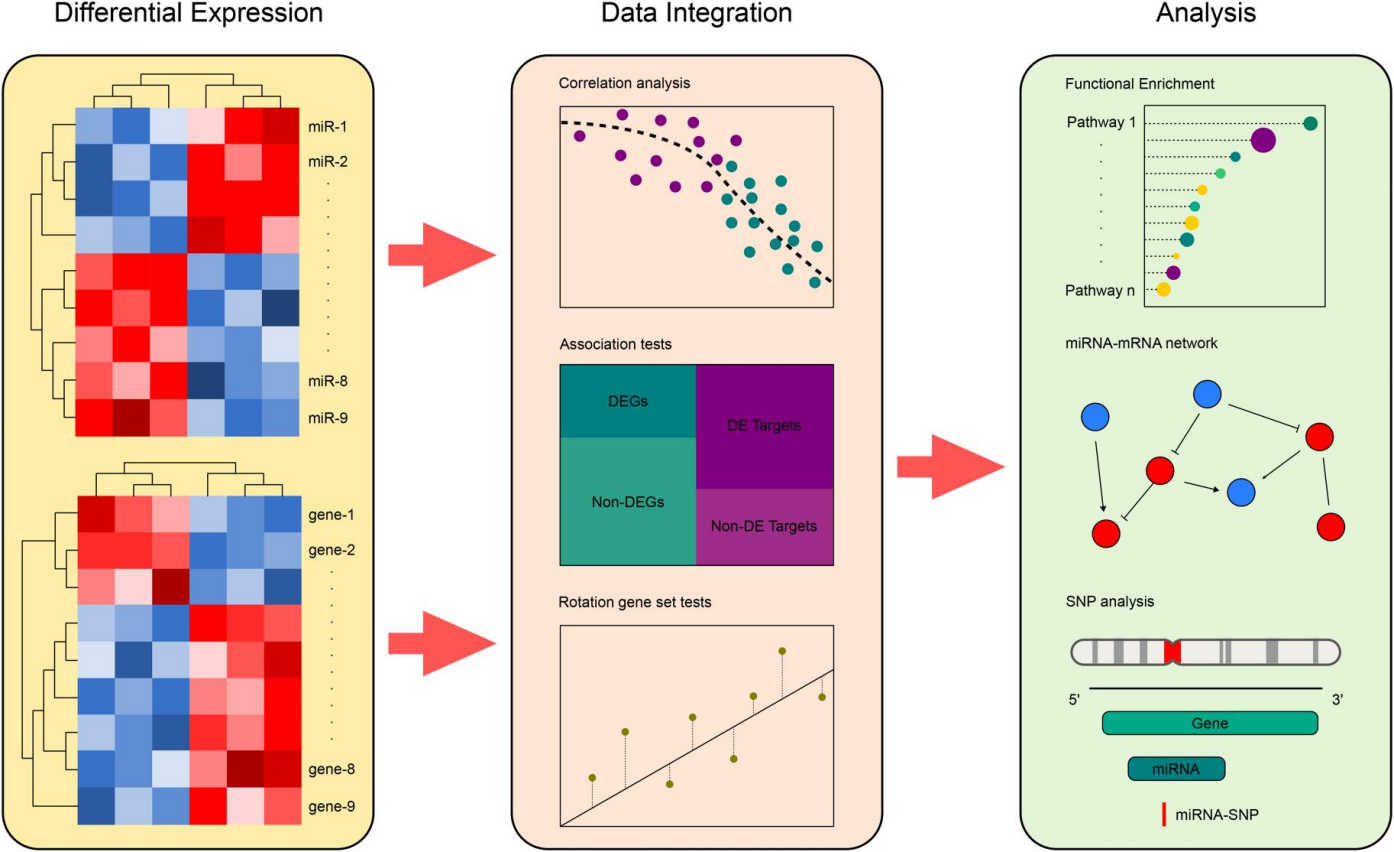
The sequential pipeline used by MIRit to integrate miRNA and mRNA expression data. After differential expression analysis, the influence of miRNAs on gene expression can be evaluated by correlation analysis when matched miRNA and mRNA expression values are available, or by association and rotation gene set tests when sample-matched measurements are lacking. Finally, MIRit allows the identification of gene sets enriched in the targets of integrated miRNAs, as well as disease-related miRNA SNPs that may influence DE-miRNAs.

An overview of the MIRit pipeline is presented in Fig. 1, while a detailed diagram of its computational workflow is shown in Fig. 1, available as supplementary data at *Bioinformatics Advances* online.

### 3.1 Benchmark of statistical approaches for integrative miRNA–mRNA analysis

The heterogeneity among available computational frameworks complicates direct and rigorous performance comparison. In particular, existing tools differ substantially in their design and intended use: some require user-provided differential expression results, whereas others integrate this step internally; some handle both microarray and RNA-Seq count data, while others support only a single data type. Moreover, strategies for miRNA–target retrieval vary widely—from automated database queries to manual user input. A detailed comparison of existing packages and their analytical workflows is provided in Table 1, available as supplementary data at *Bioinformatics Advances* online.

Given these considerations, we focused our comparison on the underlying statistical frameworks shared across most tools. Specifically, we benchmarked several statistical tests applicable to paired miRNA–mRNA datasets—Pearson’s correlation, Spearman’s correlation, partial Pearson’s correlation, and partial Spearman’s correlation—and evaluated five complementary unpaired approaches: Fisher’s exact test, Boschloo’s exact test, Fisher’s exact test with Lancaster’s mid-*P* adjustment, fry, and CAMERA.

To systematically assess their performance, we implemented a simulation-based benchmarking framework in R. This framework generated realistic paired miRNA–mRNA datasets containing known anti-correlated regulatory relationships, allowing us to test both correlation-based (paired) and categorical (unpaired) analytical strategies. Correlation-based methods were evaluated for their ability to identify true miRNA–target pairs, whereas categorical approaches were assessed for detecting miRNAs influencing gene expression. The full simulation pipeline was repeated 500 times to obtain robust estimates of performance metrics across independent synthetic datasets. Details of the simulation design and benchmarking procedure are provided in Supplementary Text 5.1–5.6, available as supplementary data at *Bioinformatics Advances* online.

**Figure 2:**
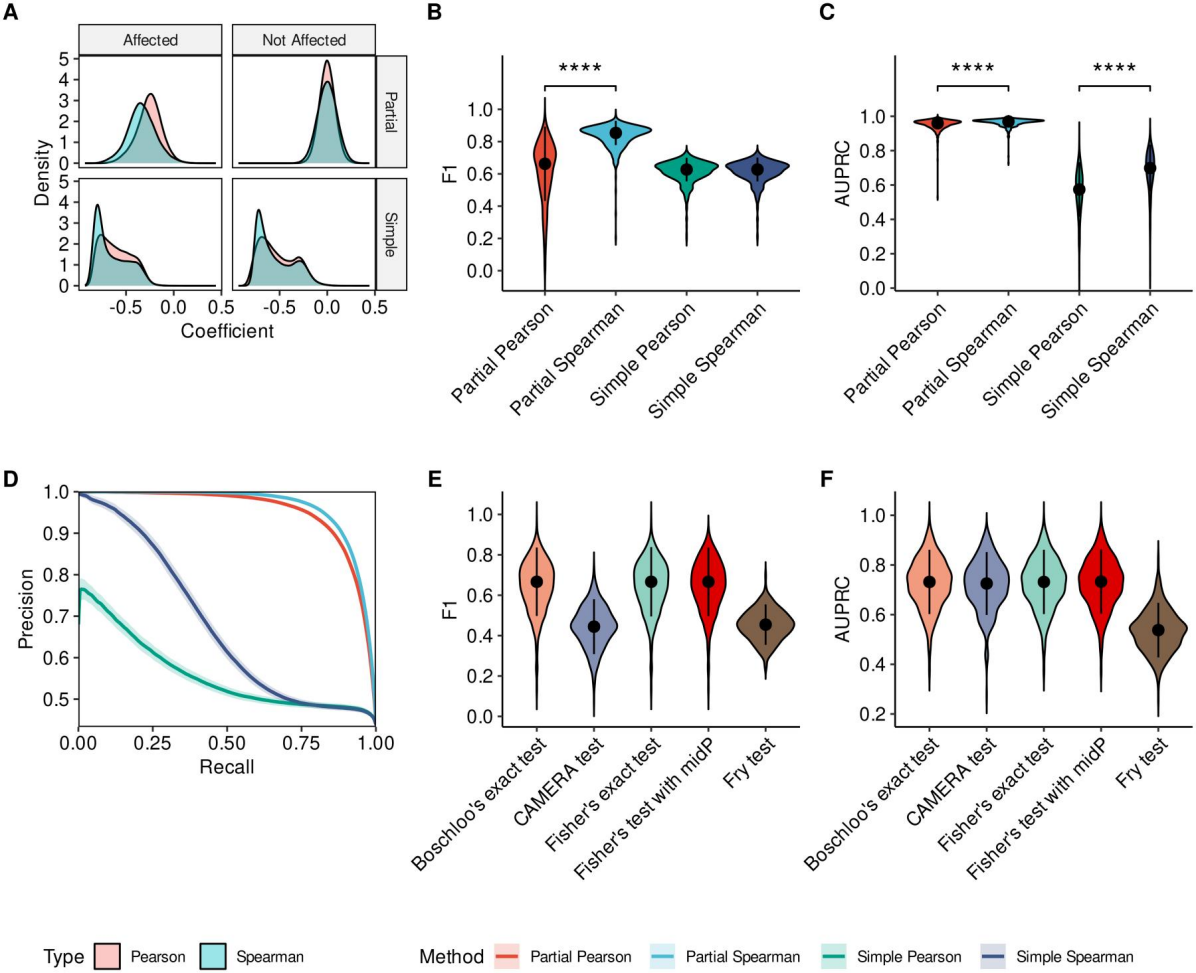
Performance of statistical approaches on simulated miRNA–mRNA datasets. A shows the distribution of correlation coefficients across 500 synthetic datasets, separated into affected and unaffected miRNA–target pairs, and shown for both simple and partial correlation analyses. B shows the mean F1 score for correlation-based approaches. C shows the mean AUPRC for correlation-based approaches. D illustrates the average precision-recall curve across simulations. E shows the average F1 score for categorical approaches. F shows the mean AUPRC for categorical approaches. ****P<0.0001.

Across simulations, we found that Spearman’s correlation consistently produced lower correlation coefficients for true miRNA–target pairs than Pearson’s correlation (Fig. 2A). This trend was observed for both simple and partial correlation analyses, with a more pronounced effect in the partial correlation case (Fig. 2A). This finding aligns with previous evidence that miRNA–target interactions are often better captured by non-linear approaches (Tian *et al.* 2024). Furthermore, we compared the F1 scores of correlation-based methods and found that partial Spearman’s correlation achieved a significantly higher F1 score (0.835±0.071) than partial Pearson’s correlation (P<0.0001; Fig. 2B). Notably, both partial correlation methods outperformed their corresponding simple correlation approaches, underscoring the benefit of adjusting for group effects that can give rise to Simpson’s paradox (Simpson 1951) and inflate false discoveries. This pattern was even more evident when comparing the area under the precision-recall curve (AUPRC) across correlation-based strategies (Fig. 2C), where partial correlation consistently yielded higher AUPRC values across simulations. In particular, Spearman’s correlation achieved the highest AUPRC among both partial and simple correlation settings (0.963±0.023 and 0.677±0.126, respectively), significantly exceeding that of Pearson’s correlation (P<0.0001; Fig. 2C). The average precision-recall curves further illustrate this trend, confirming that partial Spearman’s correlation achieved the best overall performance (Fig. 2D). For the categorical approaches, one-sided association tests achieved the highest F1 scores, with Boschloo’s exact test and Fisher’s exact test with mid-*P* adjustment performing best (0.645±0.126 and 0.646±0.126, respectively; Fig. 2E). Consistent results were observed when comparing AUPRC values (Fig. 2F).

We also evaluated FDR control across all tested strategies (Fig. 2A, available as supplementary data at *Bioinformatics Advances* online). In this analysis, one-sided association tests and partial correlation methods effectively maintained type I error rates at the nominal level, as indicated by the dashed line. The complete results of our benchmarking analysis are summarized in Table 2, available as supplementary data at *Bioinformatics Advances* online.

Given the superior performance of partial correlation analyses, we next assessed how their power depends on sample size. To this end, we repeated the benchmark using increasing sample sizes and monitored changes in F1 scores (Supplementary Text 5.7, available as supplementary data at *Bioinformatics Advances* online). As shown in Fig. 2B, available as supplementary data at *Bioinformatics Advances* online, partial correlation methods performed poorly at low sample sizes but improved markedly as the number of samples increased, reaching performance comparable to simple correlation analyses at around 25 samples per group. In contrast, the F1 scores of simple correlation methods remained relatively stable across sample sizes.

### 3.2 MiRNA–mRNA circuit profiling in DCM

DCM is a myocardial disease characterized by dilation and impaired systolic function of one or both ventricles, leading to heart failure symptoms such as dyspnea and fatigue. The etiology of the condition may be primary, attributable to genetic, inflammatory, or idiopathic conditions, or secondary to identifiable injuries to the myocardium. In this regard, ischemic DCM (ICM) and volume overload DCM (VCM) are two of the most common etiologies of the dilated-ventricle phenotype that defines DCM. Specifically, ICM refers to ventricular dilation and systolic dysfunction resulting from chronic myocardial ischemia or infarction due to coronary artery disease. Conversely, VCM arises when chronic high preload, often due to valvular regurgitation, leads to eccentric hypertrophy, chamber dilation, and eventual systolic dysfunction resembling the DCM phenotype (Bonet *et al.* 2024).

**Figure 3:**
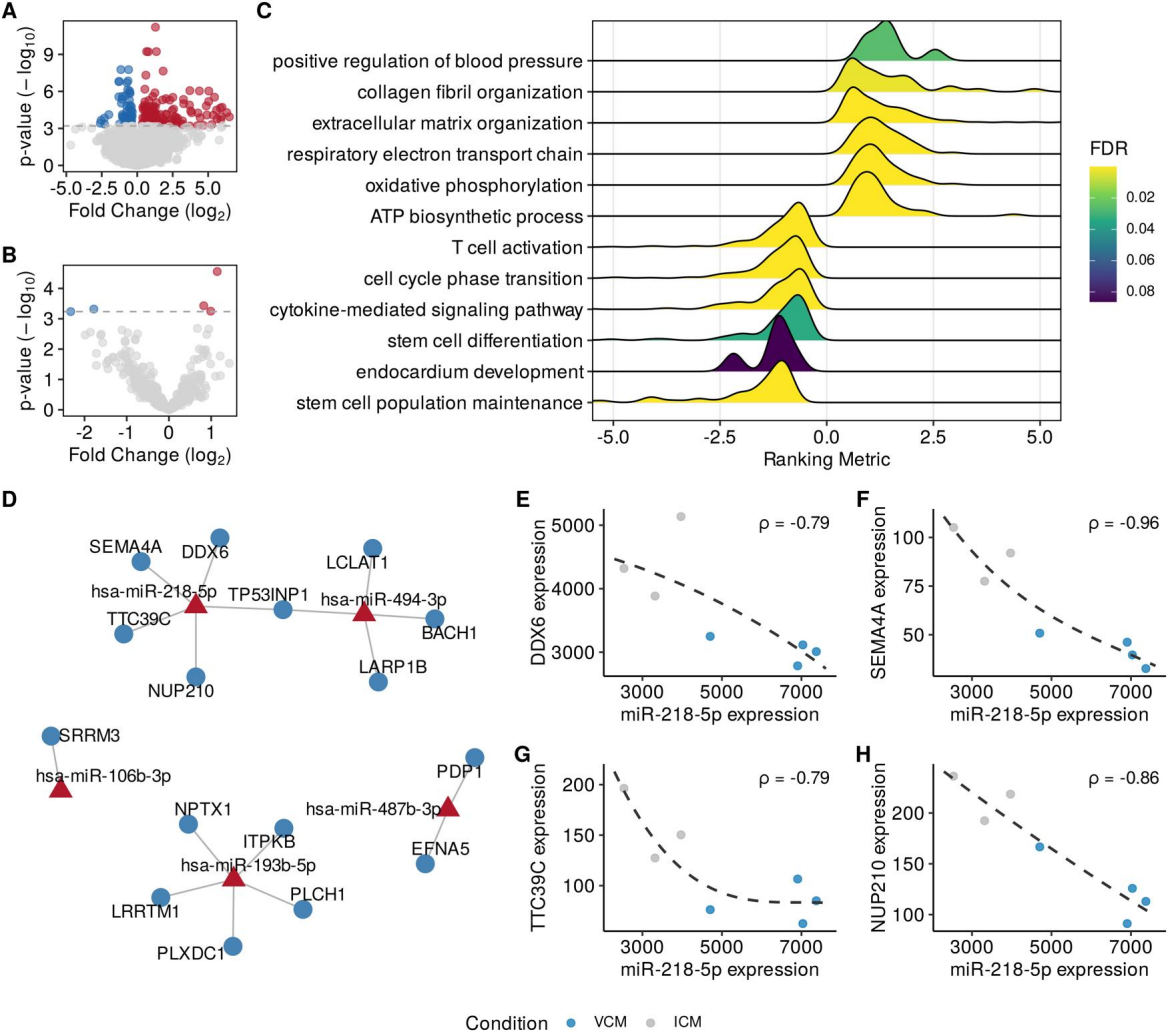
Integrative miRNA–mRNA analysis in DCM. A and B show the differentially expressed genes and miRNAs, respectively. C reports the GO biological processes that are differentially active between VCM and ICM individuals according to GSEA. D illustrates a miRNA–mRNA interaction network with the strongest interactions (ρ<−0.75). E–H show the anti-correlation between the expression levels of miR-218-5p and DDX6, SEMA4A, TTC39C, and NUP210.

In this initial case study, MIRit was employed to disentangle the miRNA regulatory networks that are active in VCM and ICM samples to identify the interactions specific to each etiology. To this end, we used a paired miRNA-Seq and RNA-Seq experiment from Bonet *et al.* (2024) of myocardial samples from 4 patients with VCM and 4 patients with ICM. After data pre-processing, differential expression analysis was performed separately for genes and miRNAs. As a result, 203 DEGs and 5 DE-miRNAs were identified (Storey’s q<0.05, Fig. 3A and B) between VCM and ICM samples. Next, GSEA was performed to investigate the functional differences between the two etiologies. As demonstrated in Fig. 3C, VCM samples had a higher expression of genes implicated in extracellular matrix organization, mitochondrial respiration, and blood pressure regulation. In contrast, a negative enrichment of pathways associated with immune response, stem cell function, and heart development was observed, suggesting that these pathways may be more active in ICM. Subsequently, we used MIRit to identify the miRNA–target pairs that were significantly anti-correlated. Following this analysis, we identified 27 interactions with a Spearman’s correlation coefficient ρ<−0.5 and a Benjamini-Hochberg adjusted *P*-value of less than 0.1 (Table 3, available as supplementary data at *Bioinformatics Advances* online). A miRNA–mRNA network with the most influential interactions (ρ<−0.75) is presented in Fig. 3D. Among the group of influential miRNAs that demonstrate differential activity in VCM and ICM subjects, miR-218-5p exhibits the highest number of anti-correlated targets, including DDX6, SEMA4A, TTC39C, and NUP210 (Fig. 3E–H). Interestingly, the differential expression of miR-218-5p and its negative correlation with DDX6, SEMA4A, and TTC39C were experimentally confirmed by the authors of the study (Bonet *et al.* 2024).

### 3.3 Integrative miRNA–mRNA analysis in ccRCC

**Figure 4:**
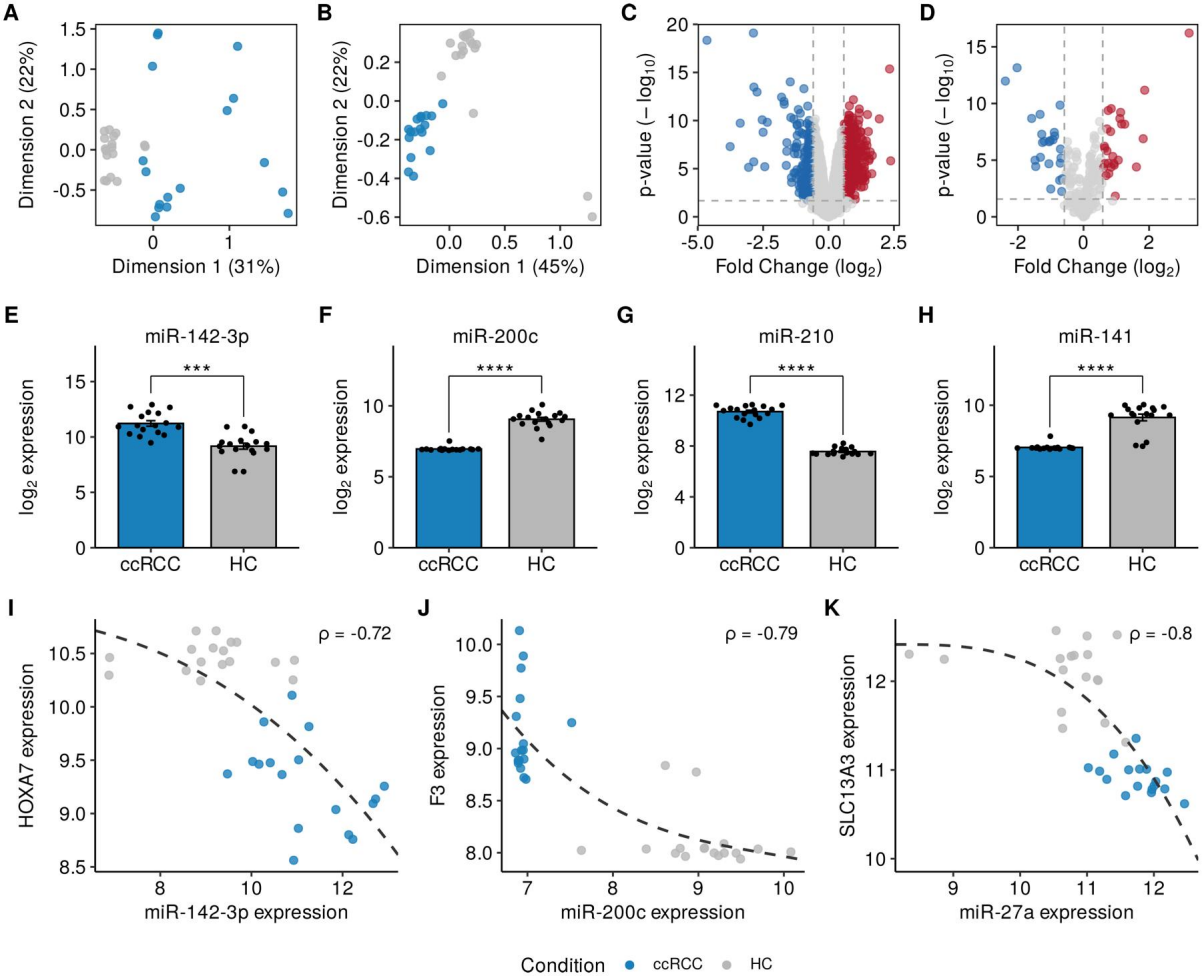
MiRNA and mRNA dysregulation in ccRCC. A and B show mRNA and miRNA expression profiles, respectively, in multidimensional space. C and D represent the results of mRNA and miRNA differential expression as volcano plots. E–H show the differential expression of miR-142-3p, miR-200c, miR-210, and miR-141, respectively. I–K show the anti-correlation observed between the expression levels of miR-142-3p and HOXA7, miR-200c and F3, and miR-27a and SLC13A3. ***P<0.001; ****P<0.0001.

The MIRit pipeline was also used to reconstruct the perturbed miRNA regulatory networks in ccRCC, the most common subtype of kidney cancer, accounting for approximately 70%–80% of cases (Rini *et al.* 2009). To investigate the role of miRNA dysregulation in the pathophysiology of ccRCC, we used a paired miRNA–mRNA experiment in which 17 ccRCC samples and 17 corresponding non-tumor samples from the same subjects were profiled by microarray technology (Liu *et al.* 2010). Upon pre-processing, we observed marked differences between ccRCC and control samples for both gene and miRNA expression, as evidenced by the multidimensional scaling (MDS) plots in Fig. 4A and B. This was further corroborated by differential expression analysis, which reported 791 DEGs and 50 DE-miRNAs with a Benjamini-Hochberg adjusted p-value less than 0.05 and an absolute fold-change greater than 1.5 (Fig. 4C and D). Notably, among the DE-miRNAs, we observed an increased expression of miR-142-3p (Fig. 4E), which is a known oncogenic miRNA in renal cell carcinoma (Li et al. 2016) and whose expression is correlated with poor prognosis (Peng *et al.* 2019, Zhang *et al.* 2023). Furthermore, the downregulation of miR-200c was detected (Fig. 4F). This miRNA acts as a tumor suppressor in ccRCC (Wang *et al.* 2015), and its expression is inversely associated with survival and disease recurrence (Saleeb *et al.* 2019). In addition, the overexpression of miR-210 and the downregulation of miR-141 were also identified (Fig. 4G and H). Once again, the upregulation of miR-210 has already been reported in ccRCC specimens and could be used as a marker of poor prognosis (Samaan *et al.* 2015). On the other hand, miR-141 is another member of the miR-200 family that acts as a tumor suppressor in ccRCC due to its negative regulation on proliferation and migration (Yu *et al.* 2013, Chen *et al.* 2014, Liu *et al.* 2022).

We then used MIRit to identify the miRNA–target interactions that are perturbed in ccRCC samples relative to healthy tissues. As a result, we identified 170 anti-correlated miRNA–target pairs (Spearman’s ρ<−0.5 and Benjamini-Hochberg adjusted *P*-value <0.1; Table 4, available as supplementary data at *Bioinformatics Advances* online). In this context, we observed a strong inverse correlation between miR-142-3p and HOXA7 (Fig. 4I), whose reduction in renal cell carcinoma is associated with poor overall survival (Cui *et al.* 2020). Furthermore, the expression of the tissue factor (F3) is negatively correlated with that of miR-200c (Fig. 4J). The observed overexpression of F3 has already been reported in renal cell carcinoma (Li and Yang 2024), and its expression was an important predictor of survival in ccRCC patients (Silva *et al.* 2014). Moreover, serum levels of F3 are dramatically reduced in ccRCC patients after surgery (Silva *et al.* 2018). Ultimately, a significant anti-correlation was observed between the expression levels of miR-27a and those of SLC13A3 (Fig. 4K). This may explain the observed downregulation of SLC13A3, which was experimentally confirmed in an independent study (Schrödter *et al.* 2016). A miRNA–mRNA network with the strongest anti-correlated interactions (Spearman’s ρ<−0.7) is presented in Fig. 3A, available as supplementary data at *Bioinformatics Advances* online. Finally, we evaluated the functional consequences of miRNA dysregulation by enrichment analysis of anti-correlated target genes. As evident in Fig. 3B, available as supplementary data at *Bioinformatics Advances* online, the target genes affected by miRNA dysregulation are highly relevant in the context of ccRCC pathophysiology. For instance, several target genes are involved in epithelial development and morphogenesis. Besides, miRNA dysregulation may induce the downregulation of genes required for cell-matrix adhesion, thereby promoting invasiveness and metastasis formation. In addition, the decreased expression of target genes belonging to kidney-related categories may be explained by the less differentiated state of ccRCC cells compared to adjacent healthy tissue. This is further supported by the depletion of genes involved in sodium and ion absorption, a phenomenon that has previously been observed in ccRCC (Zheng *et al.* 2023).

### 3.4 Unpaired miRNA–mRNA analysis in AD

**Figure 5:**
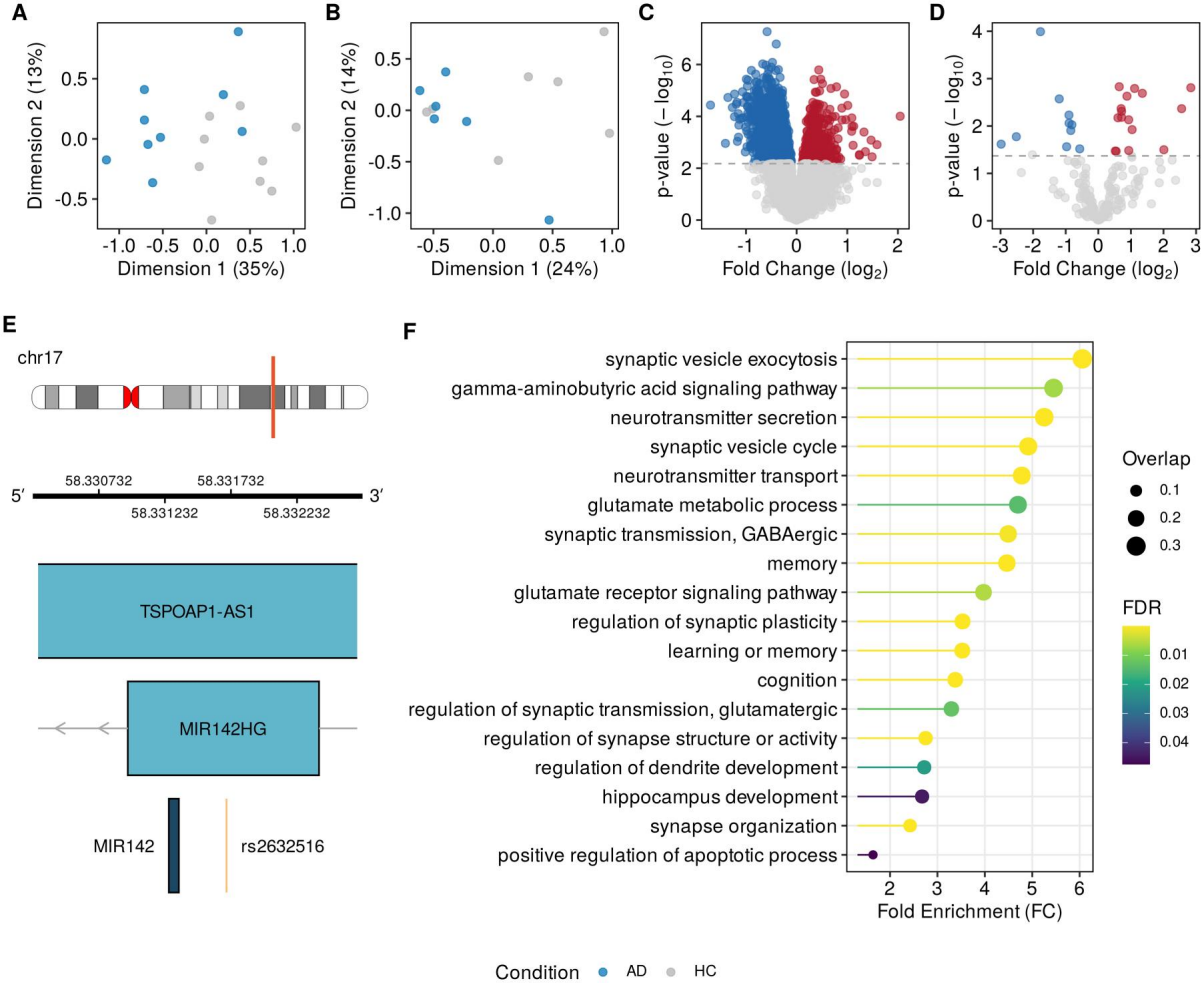
MiRNA dysregulation in the BA9 region of AD patients. A and B display the transcriptional variability in the multidimensional space of mRNAs and miRNAs, respectively, in AD patients. C and D represent mRNA and miRNA differential expression as volcano plots. E illustrates the genomic positions of rs2632516 and the locus that encodes miR-142. F shows the biological processes enriched in the down-regulated target genes affected by miRNA dysregulation.

In our third case study, we demonstrate MIRit’s capabilities in evaluating the impact of miRNA dysregulation on the prefrontal cortex of AD patients. For this study, we used two distinct datasets, one that profiled miRNAs and one that profiled mRNAs. To minimize expression variability and explore network dysregulations specific to AD pathology, we restricted our analysis to studies focusing solely on the BA9 region. Initially, we generated MDS plots for the miRNA and mRNA datasets to confirm AD-related transcriptional variation (Fig. 5A and B). Next, we performed differential expression analysis and identified 2,053 DEGs (Benjamini-Hochberg adjusted *P*-value <0.05) and 26 DE-miRNAs (Storey’s *q*-value <0.1), as shown in Fig. 5C and D. At this point, we used the findMirnaSNPs function in MIRit to identify potential AD-related SNPs within the genomic loci of DE-miRNAs. As a result, we identified the presence of rs2632516, a variant associated with increased AD susceptibility, within the host gene of miR-142, a previously identified DE-miRNA. Specifically, this polymorphism is located upstream of the miR-142 gene on the negative strand (Fig. 5E), and it may play a role in the transcriptional regulation of this miRNA. In this regard, Latini *et al.* (2021) showed that the rs2632516 variant allele is significantly associated with diminished miR-142 expression in peripheral blood mononuclear cells from patients with systemic lupus erythematosus (SLE). This is noteworthy considering that miR-142 is the second most downregulated miRNA in the BA9 region of AD patients. However, further research is needed to determine the functional significance of this polymorphism in AD pathophysiology.

Next, we collected putative miRNA targets and, since this analysis relied on unpaired datasets, we performed Boschloo’s exact test to estimate the association between miRNA and mRNA expression changes. Afterward, we discovered a statistically significant association between 18 miRNAs and 940 differentially expressed target genes with an FDR-adjusted *P*-value below 0.1 (Table 5, available as supplementary data at *Bioinformatics Advances* online). This result underscores the substantial influence of miRNA dysregulation on gene expression alterations in the prefrontal cortex of AD patients, revealing that over 45% of DEGs are targeted by at least one DE-miRNA. Finally, we performed an ORA of the dysregulated target genes associated with miRNA expression (FDR-adjusted *P*-value <0.05). As illustrated in Fig. 5F, this analysis compellingly demonstrates significant impairment of pathways central to AD pathology, including genes involved in memory, learning, and cognition—key hallmarks of the disease—highlighted through the integrative miRNA–mRNA approach. The downregulation of targets related to glutamatergic and GABAergic neurotransmission further supports the link to neurodegenerative processes. This is also reflected by the downregulation of target genes involved in synaptic plasticity, synaptic maintenance, and dendrite development. Notably, the identified pathways extend beyond the current literature in this field, emphasizing the potential of the integrative approach to uncover novel mechanisms involved in AD. While these findings are promising, independent experimental validation would strengthen the conclusions, particularly for pathways not yet characterized in previous studies. Overall, the approach exemplifies the power of integrative bioinformatics in uncovering disease-relevant pathways, fostering hypotheses for future experimental validation.

## 4 Conclusion

Since their discovery, significant efforts have been made to characterize and describe miRNAs in pathological conditions. Although omic technologies grant us the ability to measure variations in the expression of small transcripts, identifying their roles in disease mechanisms remains an unsolved problem. One effective way to evaluate the role of miRNAs in biological systems is to assess their influence on their recognized targets. However, while this strategy is conceptually sound, current approaches often rely on outdated and inappropriate procedures that severely limit the identification of disease-related mechanisms. Furthermore, the paucity of specific frameworks for integrative miRNA–mRNA analyses results in poorly reproducible conclusions as well as a defective understanding of miRNA functions. Among the poor practices commonly found in miRNA research, the use of different prediction algorithms and the heterogeneity of the criteria used to define predicted targets produces a wide range of results that are often difficult to replicate. Furthermore, the methodologies often employed to integrate miRNA and mRNA expression levels are not always appropriate given the non-linear nature of miRNA–target interactions. Finally, the lack of statistical frameworks for integrating unpaired miRNA and mRNA data dramatically hinders the derivation of biologically meaningful insights from published resources in which miRNA and mRNA expression have been evaluated in different cohorts of samples.

In this work, we introduce MIRit, a novel R package that facilitates the comprehensive characterization of impaired miRNA–mRNA networks, ranging from the identification of differentially expressed features to the reconstruction of perturbed regulatory networks. With regard to target retrieval, MIRit relies on mirDIP for obtaining plausible interactions between miRNAs and their targets based on the aggregation of multiple databases, and on miRTarBase for defining the collection of experimentally validated interactions. After identifying target genes, MIRit applies distinct statistical approaches to uncover dysregulated miRNA–mRNA interactions on the basis of available datasets. For paired data, MIRit defaults to using Spearman’s correlation analysis. In contrast, for unpaired data, MIRit supports one-sided association tests, including the Boschloo’s test, and rotation gene set tests. Apart from these core functions, MIRit provides convenient tools for identifying disease-related SNPs that may affect miRNA expression and for finding enriched categories among target genes influenced by miRNA dysregulation.

In this study, we evaluated the statistical foundations underlying integrative miRNA–mRNA analysis rather than benchmarking entire frameworks, as existing tools differ widely in input requirements, data types, and often depend on user-selected external target prediction resources, hindering direct comparison. Across 500 simulations, partial correlation methods consistently outperformed simple correlations, with partial Spearman’s correlation showing the best overall performance and aligning with the non-linear nature of miRNA–target interactions. Among unpaired approaches, Boschloo’s exact test and Fisher’s exact test with mid-*P* adjustment achieved the highest accuracy, offering practical alternatives when paired data are unavailable. Both one-sided association tests and partial correlation methods effectively controlled false discoveries, while the latter showed reduced power at small sample sizes.

Moreover, we evaluated the performance of the MIRit pipeline in three miRNA–mRNA analyses involving complex human disorders. First, we used MIRit to reveal dysregulated miRNA–target interactions in DCM. In this regard, MIRit successfully identified the anti-correlation between the expression of miR-218-5p and that of DDX6, SEMA4A, and TTC39C, a finding that was experimentally validated by Bonet *et al.* (2024) through a dual-luciferase reporter assay. Furthermore, we used MIRit to describe the miRNA–mRNA interplay in ccRCC development. After identifying DEGs and DE-miRNAs, MIRit unveiled 170 anti-correlated pairs, including miR-142-3p/HOXA7, miR-200c/F3, and miR-27a/SLC13A3. Interestingly, anti-correlated miRNA targets were enriched in crucial biological processes involved in ccRCC, including epithelial development, cell adhesion, sodium absorption, and ion homeostasis. Lastly, we examined the miRNA–target interactions disrupted in the prefrontal cortex of AD patients using unpaired miRNA and mRNA expression data. Following the identification of DEGs and DE-miRNAs, we used Boschloo’s exact test to determine which target genes were significantly affected by miRNA dysregulation and to infer which molecular processes were influenced by miRNAs. This analysis revealed the pivotal role of miRNAs in orchestrating neurodegenerative processes in the BA9 region of AD patients, as evidenced by the depletion of miRNA target genes implicated in neurotransmitter secretion, synaptic maintenance, synapse organization, memory, and cognition. Nevertheless, the involvement of the selected miRNAs in AD needs to be validated through functional experiments.

Although MIRit was showcased in DCM, ccRCC, and AD, its design is broadly applicable beyond these settings. MIRit does not rely on disease-specific priors but integrates miRNA and mRNA expression data through a fully data-driven approach. Its capacity to handle both paired and unpaired datasets allows the integration of heterogeneous data from independent cohorts or platforms. The consistent performance across diseases with distinct molecular architectures—solid tumor, neurodegeneration, and cardiac dysfunction—suggests that MIRit captures fundamental principles of miRNA-mediated regulation rather than context-specific patterns. Therefore, MIRit can be readily applied to other biological systems where miRNA–mRNA relationships are of interest. Future work may extend its scope to single-cell, time-series, or multi-omics data to further enhance its generalizability and biological interpretability.

In conclusion, we demonstrated that MIRit can be a valuable tool for researchers investigating the effects of miRNAs on gene expression by leveraging accurate resources and appropriate statistical methodologies. MIRit is entirely open source and has been made freely available on Bioconductor.

## Supplementary Material

vbag042_Supplementary_Data

## Data Availability

MIRit can be installed from Bioconductor (https://bioconductor.org/packages/release/bioc/html/MIRit.html), and its source code is available on GitHub (https://github.com/jacopo-ronchi/MIRit). The datasets used in this study are publicly available on GEO under the accession numbers GSE243406, GSE16441, GSE63501, and GSE150696. The code used to generate all results and conclusions of this study is available on Github (https://github.com/jacopo-ronchi/MIRit_supporting_files) and is archived on Zenodo (https://doi.org/10.5281/zenodo.17493903). Extensive documentation for using MIRit is available on the Bioconductor package page and at the MIRit documentation website (https://jacopo-ronchi.github.io/MIRit/).

## References

[vbag042-B1] Aleksander SA , Balhoff J, Carbon S et al; The Gene Ontology Consortium. The gene ontology knowledgebase in 2023. Genetics 2023;224:iyad031. 10.1093/genetics/iyad031 36866529 PMC10158837

[vbag042-B2] Ardekani AM , Naeini MM. The role of microRNAs in human diseases. AJMB 2010;2:161–79. https://www.ncbi.nlm.nih.gov/pmc/articles/PMC3558168/23407304 PMC3558168

[vbag042-B3] Bonet F , Hernandez-Torres F, Ramos-Sánchez M et al Unraveling the etiology of dilated cardiomyopathy through differential miRNA–mRNA interactome. Biomolecules 2024;14:524. 10.3390/biom14050524 38785931 PMC11117812

[vbag042-B4] Boyle EI , Weng S, Gollub J et al GO::TermFinder—open source software for accessing gene ontology information and finding significantly enriched gene ontology terms associated with a list of genes. Bioinformatics 2004;20:3710–5. 10.1093/bioinformatics/bth456 15297299 PMC3037731

[vbag042-B5] Carvalho BS , Irizarry RA. A framework for oligonucleotide microarray preprocessing. Bioinformatics 2010;26:2363–7. 10.1093/bioinformatics/btq431 20688976 PMC2944196

[vbag042-B6] Cava C , Colaprico A, Bertoli G et al SpidermiR: an R/bioconductor package for integrative analysis with miRNA data. Int J Mol Sci 2017;18:274. 10.3390/ijms18020274 28134831 PMC5343810

[vbag042-B7] Chen X , Wang X, Ruan A et al miR-141 is a key regulator of renal cell carcinoma proliferation and metastasis by controlling EphA2 expression. Clin Cancer Res 2014;20:2617–30. 10.1158/1078-0432.CCR-13-3224 24647573

[vbag042-B8] Clough E , Barrett T. The gene expression omnibus database. In: Mathé E, Davis S (eds.), Statistical Genomics: Methods and Protocols. New York, NY: Springer, 2016, 93–110. 10.1007/978-1-4939-3578-9_5 PMC4944384 27008011

[vbag042-B9] Cui S , Yu S, Huang H-Y et al miRTarBase 2025: updates to the collection of experimentally validated microRNA–target interactions. Nucleic Acids Res 2025;53:D147–56. 10.1093/nar/gkae1072 39578692 PMC11701613

[vbag042-B10] Cui Y , Yan M, Zhang C et al Comprehensive analysis of the HOXA gene family identifies HOXA13 as a novel oncogenic gene in kidney renal clear cell carcinoma. J Cancer Res Clin Oncol 2020;146:1993–2006. 10.1007/s00432-020-03259-x 32444962 PMC11804755

[vbag042-B11] Friedman RC , Farh KK-H, Burge CB et al Most mammalian mRNAs are conserved targets of microRNAs. Genome Res 2009;19:92–105. 10.1101/gr.082701.108 18955434 PMC2612969

[vbag042-B12] Gillespie M , Jassal B, Stephan R et al The reactome pathway knowledgebase 2022. Nucleic Acids Res 2022;50:D687–92. 10.1093/nar/gkab1028 34788843 PMC8689983

[vbag042-B13] Hauschild A-C , Pastrello C, Ekaputeri G et al MirDIP 5.2: tissue context annotation and novel microRNA curation. Nucleic Acids Res 2023;51:D217–25. 10.1093/nar/gkac1070 36453996 PMC9825511

[vbag042-B14] Kanehisa M , Goto S. KEGG: kyoto encyclopedia of genes and genomes. Nucleic Acids Res 2000;28:27–30. 10.1093/nar/28.1.27 10592173 PMC102409

[vbag042-B15] Latini A , Ciccacci C, Benedittis GD et al Altered expression of miR-142, miR-155, miR-499a and of their putative common target MDM2 in systemic lupus erythematosus. Epigenomics 2021;13:5–13. https://www.futuremedicine.com/doi/10.2217/epi-2020-0278 33337917 10.2217/epi-2020-0278

[vbag042-B16] Law CW , Chen Y, Shi W et al Voom: precision weights unlock linear model analysis tools for RNA-seq read counts. Genome Biol 2014;15:R29. 10.1186/gb-2014-15-2-r29 24485249 PMC4053721

[vbag042-B17] Le TD , Zhang J, Liu L et al miRLAB: an R based dry lab for exploring miRNA-mRNA regulatory relationships. PLoS One 2015;10:e0145386. https://journals.plos.org/plosone/article?id=10.1371/journal.pone.0145386 26716983 10.1371/journal.pone.0145386 PMC4696828

[vbag042-B18] Li Y , Yang Y. Label-free quantitative proteomics reveals the mechanisms of aurora kinase B in renal cell carcinoma. SAGE Open Med 2024;12:20503121241228474. 10.1177/20503121241228474 38516642 PMC10956137

[vbag042-B19] Li Y , Chen D, Jin L et al Oncogenic microRNA-142-3p is associated with cellular migration, proliferation and apoptosis in renal cell carcinoma. Oncol Lett 2016;11:1235–41. https://www.ncbi.nlm.nih.gov/pmc/articles/PMC4734216/26893725 10.3892/ol.2015.4021 PMC4734216

[vbag042-B20] Liu H , Brannon AR, Reddy AR et al Identifying mRNA targets of microRNA dysregulated in cancer: with application to clear cell renal cell carcinoma. BMC Syst Biol 2010;4:51. 10.1186/1752-0509-4-51 20420713 PMC2876063

[vbag042-B21] Liu Y , Fu W, Yin F et al miR-141-3p suppresses development of clear cell renal cell carcinoma by regulating NEK6. Anticancer Drugs 2022;33:e125–33. https://journals.lww.com/anti-cancerdrugs/abstract/2022/01000/mir_141_3p_suppresses_development_of_clear_cell.30.aspx 34387594 10.1097/CAD.0000000000001158

[vbag042-B22] Love MI , Huber W, Anders S. Moderated estimation of fold change and dispersion for RNA-seq data with DESeq2. Genome Biol 2014;15:550. 10.1186/s13059-014-0550-8 25516281 PMC4302049

[vbag042-B23] Low CYB , Lee JH, Lim FTW et al Isoform-specific upregulation of FynT kinase expression is associated with tauopathy and glial activation in Alzheimer’s disease and Lewy body dementias. Brain Pathol 2021;31:253–66. https://onlinelibrary.wiley.com/doi/abs/10.1111/bpa.12917 33128789 10.1111/bpa.12917 PMC8017997

[vbag042-B24] Magno R , Maia A-T. Gwasrapidd: an R package to query, download and wrangle GWAS catalog data. Bioinformatics 2020;36:649–50. 10.1093/bioinformatics/btz605 31373609 PMC9883700

[vbag042-B25] Martens M , Ammar A, Riutta A et al WikiPathways: connecting communities. Nucleic Acids Res 2021;49:D613–21. 10.1093/nar/gkaa1024 33211851 PMC7779061

[vbag042-B26] Movassagh M , Morton SU, Hehnly C et al mirTarRnaSeq: an R/bioconductor statistical package for miRNA-mRNA target identification and interaction analysis. BMC Genomics 2022;23:439. 10.1186/s12864-022-08558-w 35698050 PMC9191533

[vbag042-B27] O’Brien J , Hayder H, Zayed Y et al Overview of microRNA biogenesis, mechanisms of actions, and circulation. Front Endocrinol (Lausanne) 2018;9:402. https://www.frontiersin.org/articles/10.3389/fendo.2018.00402 30123182 10.3389/fendo.2018.00402 PMC6085463

[vbag042-B28] Patel K , Chandrasegaran S, Clark IM et al TimiRGeN: r /bioconductor package for time series microRNA–mRNA integration and analysis. Bioinformatics 2021;37:3604–9. 10.1093/bioinformatics/btab377 33993215 PMC8545325

[vbag042-B29] Patil AH , Halushka MK. miRge3.0: a comprehensive microRNA and tRF sequencing analysis pipeline. NAR Genom Bioinform 2021;3:lqab068. 10.1093/nargab/lqab068 34308351 PMC8294687

[vbag042-B30] Peng X , Pan X, Liu K et al miR-142-3p as a novel biomarker for predicting poor prognosis in renal cell carcinoma patients after surgery. Int J Biol Markers 2019;34:302–8. 10.1177/1724600819866456 31378131

[vbag042-B31] Rini BI , Campbell SC, Escudier B. Renal cell carcinoma. Lancet 2009;373:1119–32. 10.1016/S0140-6736(09)60229-4 19269025

[vbag042-B32] Ritchie ME , Phipson B, Wu D et al Limma powers differential expression analyses for RNA-sequencing and microarray studies. Nucleic Acids Res 2015;43:e47. 10.1093/nar/gkv007 25605792 PMC4402510

[vbag042-B33] Robinson MD , McCarthy DJ, Smyth GK. edgeR: a bioconductor package for differential expression analysis of digital gene expression data. Bioinformatics 2010;26:139–40. 10.1093/bioinformatics/btp616 19910308 PMC2796818

[vbag042-B34] Saleeb R , Kim SS, Ding Q et al The miR-200 family as prognostic markers in clear cell renal cell carcinoma. Urol Oncol 2019;37:955–63. https://www.sciencedirect.com/science/article/pii/S1078143919303266 31635993 10.1016/j.urolonc.2019.08.008

[vbag042-B35] Samaan S , Khella HWZ, Girgis A et al miR-210 is a prognostic marker in clear cell renal cell carcinoma. J Mol Diagn 2015;17:136–44. https://www.sciencedirect.com/science/article/pii/S1525157814002463 25555365 10.1016/j.jmoldx.2014.10.005

[vbag042-B36] Santa-Maria I , Alaniz ME, Renwick N et al Dysregulation of microRNA-219 promotes neurodegeneration through post-transcriptional regulation of tau. J Clin Invest 2015;125:681–6. https://www.jci.org/articles/view/78421 25574843 10.1172/JCI78421 PMC4319412

[vbag042-B37] Schrödter S , Braun M, Syring I et al Identification of the dopamine transporter SLC6A3 as a biomarker for patients with renal cell carcinoma. Mol Cancer 2016;15:10. 10.1186/s12943-016-0495-5 26831905 PMC4736613

[vbag042-B38] Silva DD , Noronha JAP, da Silva VD et al Increased tissue factor expression is an independent predictor of mortality in clear cell carcinoma of the kidney. Int Braz J Urol 2014;40:499–506. 10.1590/S1677-5538.IBJU.2014.04.08 25251954

[vbag042-B39] Silva DD , Noronha JAP, da Costa BEP et al Serum tissue factor as a biomarker for renal clear cell carcinoma. Int Braz J Urol 2018;44:38–44. https://www.scielo.br/j/ibju/a/Mv3ptdbrgMxHM3kTngtfzxL/?lang=en 28727370 10.1590/S1677-5538.IBJU.2017.0007 PMC5815530

[vbag042-B40] Simpson EH. The interpretation of interaction in contingency tables. J R Stat Soc Ser B Stat Methodol 1951;13:238–41. 10.1111/j.2517-6161.1951.tb00088.x

[vbag042-B41] Sollis E , Mosaku A, Abid A et al The NHGRI-EBI GWAS catalog: knowledgebase and deposition resource. Nucleic Acids Res 2023;51:D977–85. 10.1093/nar/gkac1010 36350656 PMC9825413

[vbag042-B42] Storey JD. A direct approach to false discovery rates. J R Stat Soc Ser B Stat Methodol 2002;64:479–98. 10.1111/1467-9868.00346

[vbag042-B43] Subramanian A , Tamayo P, Mootha VK et al Gene set enrichment analysis: a knowledge-based approach for interpreting genome-wide expression profiles. Proc Natl Acad Sci USA 2005;102:15545–50. https://www.pnas.org/doi/10.1073/pnas.0506580102 16199517 10.1073/pnas.0506580102 PMC1239896

[vbag042-B44] Tian S , Zhao Z, Ren B et al Non-linear relationship between MiRNA regulatory activity and binding site counts on target mRNAs. Data (Basel) 2024;9:111. https://www.mdpi.com/2306-5729/9/10/111

[vbag042-B45] Vila-Casadesús M , Gironella M, Lozano JJ. MiRComb: an R package to analyse miRNA-mRNA interactions. Examples across five digestive cancers. PLoS One 2016;11:e0151127. https://journals.plos.org/plosone/article?id=10.1371/journal.pone.0151127 26967326 10.1371/journal.pone.0151127 PMC4788200

[vbag042-B46] Wang T-T , Lee C-Y, Lai L-C et al anamiR: integrated analysis of microRNA and gene expression profiling. BMC Bioinform 2019;20:239. 10.1186/s12859-019-2870-x PMC6518761 31088348

[vbag042-B47] Wang X , Chen X, Han W et al miR-200c targets CDK2 and suppresses tumorigenesis in renal cell carcinoma. Mol Cancer Res 2015;13:1567–77. 10.1158/1541-7786.MCR-15-0128 26248649

[vbag042-B48] Wu D , Smyth GK. Camera: a competitive gene set test accounting for inter-gene correlation. Nucleic Acids Res 2012;40:e133. 10.1093/nar/gks461 22638577 PMC3458527

[vbag042-B49] Wu D , Lim E, Vaillant F et al ROAST: rotation gene set tests for complex microarray experiments. Bioinformatics 2010;26:2176–82. 10.1093/bioinformatics/btq401 20610611 PMC2922896

[vbag042-B50] Yu X-y , Zhang Z, Liu J et al MicroRNA-141 is downregulated in human renal cell carcinoma and regulates cell survival by targeting CDC25B. Onco Targets Ther 2013;6:349–54. https://www.dovepress.com/microrna-141-is-downregulated-in-human-renal-cell-carcinoma-and-regula-peer-reviewed-fulltext-article-OTT 23596351 10.2147/OTT.S41343 PMC3627343

[vbag042-B51] Zhang Y , Ma S, Zhang J et al MicroRNA-142-3p promotes renal cell carcinoma progression by targeting RhoBTB3 to regulate HIF-1 signaling and GGT/GSH pathways. Sci Rep 2023;13:5935. https://www.nature.com/articles/s41598-022-21447-2 37045834 10.1038/s41598-022-21447-2 PMC10097650

[vbag042-B52] Zheng Q , Wang Y, Zhao R et al Inactivation of epithelial sodium ion channel molecules serves as effective diagnostic biomarkers in clear cell renal cell carcinoma. Genes Genomics 10.1007/s13258-023-01376-8 37133722

