# Supplementary material for: MIRit: an integrative R framework for the identification of impaired miRNA–mRNA regulatory networks in complex diseases
Source: Bioinform Adv. 2026 Feb 13;6(1):vbag042. doi: 10.1093/bioadv/vbag042 (PMC12961272; doi:10.1093/bioadv/vbag042)
Supplement: vbag042_Supplementary_Data [file vbag042_supplementary_data.zip › MIRit_supplementary_text.pdf]

MIRit: an integrative R framework for the identification of impaired miRNA-mRNA regulatory networks in complex diseases

Jacopo Ronchi and Maria Foti

Supplementary Text

S1 Software's architecture **1**
S1.1 Differential expression analysis . . . 1
S1.2 Functional enrichment of dysregulated genes . . . 1
S1.3 Association of miRNAs with disease-related variants . . . 1
S1.4 MiRNA targets retrieval . . . 1
S1.5 Integration of miRNA and mRNA dysregulations . . . 2

S2 Dilated Cardiomyopathy (DCM) dataset **3**

S3 Clear cell renal cell carcinoma (ccRCC) dataset **3**

S4 Alzheimer's disease (AD) dataset **4**

S5 Benchmarking statistical tests for miRNA–mRNA integration **5**
S5.1 Simulation of paired miRNA–mRNA expression datasets . . . 5
S5.2 Modelling miRNA–mRNA regulation . . . 5
S5.3 Differential expression analysis . . . 6
S5.4 Correlation-based integration (paired analyses) . . . 6
S5.5 Categorical (unpaired) integration tests . . . 6
S5.6 Benchmark procedure . . . 7
S5.7 Comparison of F1 scores across varying sample sizes . . . 7

S1 Software's architecture

S1.1 Differential expression analysis

The initial phase of the MIRit pipeline entails the differential expression analysis of miRNAs and genes. In this regard, MIRit offers several options depending on the technology used to generate the expression data. When expression values are obtained from microarrays, MIRit uses the standard pipeline in the limma package [1] to estimate differentially expressed features. On the other hand, for RNA-Seq and miRNA-Seq experiments, MIRit offers a choice between several approaches, including edgeR [2], DESeq2 [3], and limma-voom [4]. Importantly, MIRit offers the ability to fully customize the parameters used for differential expression analysis, allowing for complex and multivariate experimental designs. In addition, users can perform and load their own analyses. This allows MIRit to process results from a variety of tools and technologies, including proteomic data analyzed using specialized pipelines.

S1.2 Functional enrichment of dysregulated genes

After performing a differential expression analysis, different approaches are typically used to determine which cellular processes are dysregulated under the given conditions of interest. In this respect, MIRit provides extensive support for functional enrichment of genes using over-representation analysis (ORA) [5], gene set enrichment analysis (GSEA) [6], and Correlation Adjusted MEan RAnk gene set test (CAMERA) [7], allowing the user to infer compromised biological functions using the preferred method. Notably, for ORA, MIRit performs enrichment separately for upregulated and downregulated genes, an approach that has been shown to be more powerful than enriching all differentially expressed genes (DEGs) [8]. With respect to gene set collections, MIRit provides support for all of the most commonly used biological resources, including Gene Ontology (GO) [9], KEGG [10], WikiPathways [11], MsigDb [6], and Reactome [12].

S1.3 Association of miRNAs with disease-related variants

MIRit provides a useful feature for examining the overlap between DE-miRNA loci and genomic variants associated with human disorders. This is relevant as single nucleotide polymorphisms (SNPs) found within miRNA gene loci can have serious implications on the biological function of these transcripts. In fact, a SNP located within a miRNA gene could alter its expression or change the spectrum of miRNA targets. Specifically, MIRit retrieves disease-associated SNPs using the gwasrapidd package [13], which directly queries the NHGRI-EBI catalog of published genome-wide association studies [14]. Next, the tool retrieves the genomic positions of DE-miRNAs from the Ensembl database [15] and overlays them with the positions of disease-associated SNPs.

S1.4 MiRNA targets retrieval

Before performing integrative miRNA-mRNA analyses, it is essential to determine the target genes of expressed miRNAs. Over the years, several resources have been developed to predict and collect miRNA-target interactions. These can be categorized into prediction databases, which contain computationally determined interactions, and validated databases, which contain only interactions proven by biomolecular experiments. Several bioinformatic pipelines prioritize validated interactions, even though they tend to be less numerous than predicted pairs. Predicted interactions, on the other hand, are more representative of the real targetome but also produce more false positives. MiRNA target prediction algorithms are also hampered

by a low degree of overlap between different tools. To overcome this challenge, one option is to consider interactions when predicted by multiple tools. However, this approach may not capture a significant number of significant associations. Alternatively, one could aggregate interactions predicted by multiple resources. Although this method identifies more true associations, it also results in a higher proportion of false discoveries. Despite these limitations, these approaches are still the most widely employed to identify miRNA-target interactions. However, an emerging strategy is to incorporate alternative statistics to rank miRNA-target predictions obtained from multiple algorithms. In this context, the microRNA Data Integration Portal (mirDIP) database [16] implements this idea by combining predictions from 24 different resources through an integrated score derived from different prediction metrics. Given the superior accuracy of this approach [16, 17], MIRit uses predicted miRNA-target interactions from the mirDIP database along with experimentally validated interactions from miRTarBase [18].

S1.5 Integration of miRNA and mRNA dysregulations

After defining miRNA-target interactions, an integrative analysis can be performed so that putative miRNA-target pairs are only considered when an inverse relationship is observed. For this purpose, a variety of statistical approaches can be used. When both miRNA and gene expression measurements are available for the same samples, a correlation analysis is the recommended procedure. The aim of this analysis is to quantify the extent to which the expression of target genes depends on the expression of their miRNA regulators. Several statistical coefficients can be used for this purpose, including Pearson's r, Spearman's ρ, and Kendall's τ_b. Pearson's r is probably the most commonly used to correlate miRNA and gene expression; however, it assumes a linear relationship, which is typically not true for miRNAs, as their interactions are characterized by imperfect complementarity. In addition, miRNAs can target multiple genes with different binding sites, suggesting that a simple linear relationship may not adequately describe the complexity of these interactions. In contrast, Spearman's and Kendall's tau-b correlation coefficients are more appropriate to describe the interplay between miRNAs and target genes because they are robust to non-linear relationships and outliers. Nevertheless, Kendall's correlation relies solely on the number of concordant and discordant pairs and is less sensitive than Spearman's correlation. Consequently, in scenarios where there are numerous ties or the sample size is limited, Kendall's correlation may exhibit reduced detection capability. Consequently, Spearman's correlation coefficient is the default measure employed in MIRit to assess the correlation between miRNA and gene expression. Notably, when unwanted technical artifacts affect gene and miRNA expression, MIRit allows the use of partial correlation analysis. This involves fitting a linear model to the expression data and then adjusting the matrices by subtracting the variation due to batch effects. In this way, correlation is performed between the residuals of the models. This is necessary because correlation analysis evaluates the concordance of measurements taken on a per-sample basis, which can be affected by confounding variables.

However, in instances where sample correspondence is lacking, a correlation analysis cannot be performed. In such cases, one-sided association tests can be used to determine whether the targets of downregulated miRNAs are statistically enriched in upregulated genes, and vice versa, if the targets of upregulated miRNAs are statistically enriched in downregulated genes. To this end, a 2x2 contingency table is constructed for each miRNA, as illustrated in **Table 1**.

The effect of each miRNA on its targets is then evaluated using a one-sided association test, such as Fisher's exact test, which yields a p-value for each miRNA. These p-values are subsequently adjusted for multiple testing. MIRit optionally applies Lancaster's mid-p correction to improve statistical power while controlling the type I error rate [19]. However, as Fisher's exact

Table 1: The 2x2 contingency table that MIRit uses for one-sided association tests.

	Genes		
	Targets	Non targets	Row total
Differentially expressed	a	b	$a+b$
Non differentially expressed	c	d	$c+d$
Column total	$a+c$	$b+d$	n

test assumes fixed row and column margins—a condition often unmet in genomic data—MIRit defaults to Boschloo's exact test, a powerful variant of Barnard's test, which accommodates variable marginal totals [20].

Finally, unpaired data can take advantage of linear models and rotation gene set tests to infer the impact of DE-miRNAs on target gene expression. The purpose of this approach is to assess whether each miRNA leads to differentially expressed targets in the opposite direction. In detail, "fry", a fast approximation of rotation gene set testing [21] implemented in the limma package, is used to measure the impact of miRNAs on changes in the expression of their target genes.

S2 Dilated Cardiomyopathy (DCM) dataset

Count matrices were retrieved from the Gene Expression Omnibus (GEO) database [22] (accession number: GSE243406) and imported into the R environment [23]. Genes and miRNAs with less than 10 reads in at least 3 samples were discarded. Subsequently, DESeq2 was used to identify DEGs and DE-miRNAs between samples with volume overload DCM (VCM) and those with ischemic DCM (ICM). P-values were then adjusted using the Storey's q-value approach [24], and features with $q < 0.05$ were deemed statistically significant. Following the differential expression analysis, GSEA was performed using the `enrichGenes` function to find the GO biological processes that are differentially active in VCM and ICM samples. Categories with a Benjamini-Hochberg adjusted p-value of less than 0.1 were considered significant. Thereafter, putative miRNA targets were obtained by merging the top 5% of predicted interactions in the mirDIP database with validated interactions included in miRTarBase. Finally, influential miRNA-target interactions were identified using Spearman's correlation analysis (Spearman's $\rho < -0.5$ and Benjamini-Hochberg adjusted p-value < 0.1).

S3 Clear cell renal cell carcinoma (ccRCC) dataset

Raw microarray data of miRNA and mRNA expression were obtained from GEO (accession number: GSE16441) [25]. The data were then imported into R, and the intensities underwent background correction and quantile normalization. The `avereps` function in limma was used to average the intensity of probes targeting the same feature. Probes that were not expressed above the background intensity, control probes, and probes without proper annotation were then excluded from the analysis. In addition, poorly expressed probes with a median intensity of less than 7 for miRNAs or 8 for genes were discarded. After data pre-processing, multidimensional scaling (MDS) was used to investigate miRNA and mRNA expression variability. Subsequently, MIRit was used for differential expression analysis, target retrieval and data integration. In particular, the limma pipeline was used to identify differentially expressed features with an absolute fold-change greater than 1.5 and a Benjamini-Hochberg adjusted p-value less than 0.05. Since this dataset includes a tumor sample and adjacent healthy tissue

from each individual donor, patient identity served as a covariate to account for the correlation between samples from the same subject. Furthermore, array weights were estimated to remove the effect of array quality across samples, and the robust parameter was used when performing the moderated t-test to limit the influence of hyper-variable probes. Following the differential expression analysis, the `getTargets` function in MIRit was used to retrieve the targets of the expressed miRNAs. Only the top 5% of predicted miRNA-target interactions in the mirDIP database were considered for each miRNA, along with validated interactions in miRTarBase. After identification of putative target genes, Spearman's correlation analysis was performed using the `mirnaIntegration` function in MIRit to identify the anti-correlated miRNA-target pairs (Spearman's $\rho < -0.5$ and Benjamini-Hochberg adjusted p-value < 0.1). Finally, the `enrichTargets` function in MIRit was used to perform an over-representation analysis of differentially expressed miRNA targets according to the gene sets provided by GO (Benjamini-Hochberg adjusted p-value < 0.1).

S4 Alzheimer's disease (AD) dataset

For the AD case study, two distinct datasets involving different subjects were used. The first dataset (GEO accession: GSE63501) consists of a small RNA-Seq experiment [26] examining postmortem tissues from the Brodmann area 9 (BA9) of 6 patients with AD, 3 subjects with tangle-predominant dementia (TPD), and 7 healthy controls. Conversely, the second dataset (GEO accession: GSE150696) employed microarray expression data of the BA9 region obtained through the Affymetrix Human Transcriptome Array 2.0, including samples from 9 AD patients, 12 patients with dementia with Lewy bodies (DLB), 12 subjects with Parkinson's disease dementia (PDD), and 9 healthy donors [27]. For the purposes of this analysis, we maintained only the expression data of AD patients and healthy subjects. With regard to the miRNA dataset, the corresponding FASTQ files were downloaded from the Sequence Read Archive (SRA) [28] using the SRA toolkit. We then used the miRge 3.0 pipeline [29] to pre-process the miRNA reads, which includes adapter trimming, quality control with Cutadapt [30], and alignment of the reads to a modified miRNA library with bowtie [31]. For adapter trimming, we used TCCGACGATC as the 5' adapter sequence and TCGTATGCCGTCTTCTGCTTGT for the 3' adapter. Information on the number of sequenced reads and identified miRNAs for each sample can be found in **Table S6**. Only miRNAs with a minimum of 10 reads observed in at least 6 subjects were included. Conversely, with regard to gene expression, we obtained the transcriptomes of both AD and control samples by downloading microarray CEL files from GEO and importing them into R via the oligo package [32]. We then used the robust multi-array average (RMA) algorithm to normalize probe expression and summarize values at the transcript level. Probes that lacked adequate annotation or exhibited a median intensity below 6 were excluded from the subsequent analysis. Subsequently, the `avereps` function in limma was used to average probes targeting the same gene. Concerning mRNAs, the limma pipeline was used to identify DEGs between AD and control samples. Notably, sex was used as a covariate in the linear model to account for sex-related effects in gene expression. In addition, microarray quality weights were estimated and incorporated into the model. After correcting for multiple testing, genes with a Benjamini-Hochberg adjusted p-value < 0.05 were considered significant. In contrast, for miRNAs, the limma-voom pipeline was used to identify DE-miRNAs between AD cases and controls. Again, quality weights were included in the model to remove the effect of variable library quality between samples. In this case, miRNAs with a Storey's adjusted q-value less than 0.1 were defined as DE-miRNAs. Next, the `findMirnaSNPs` function in MIRit was used to identify AD-associated SNPs in the NHGRI-EBI catalog that may affect the expression or functioning of the identified DE-miRNAs. Following this, MIRit was used to reconstruct the perturbed miRNA-mRNA network in the BA9 region of AD patients. To this end, the tar-

get genes of the analyzed miRNAs were obtained by considering the experimentally validated interactions in miRTarBase and the top 5% of those predicted by mirDIP. After identifying putative interactions, the Boschloo test was used to identify miRNAs that significantly affect the expression of their target genes (Benjamini-Hochberg adjusted p-value < 0.1). Finally, the functional impact of miRNA dysregulation was evaluated by over-representation analysis of the genes targeted by the influential miRNAs according to the gene sets in GO biological processes (Benjamini-Hochberg adjusted p-value < 0.1).

S5 Benchmarking statistical tests for miRNA–mRNA integration

S5.1 Simulation of paired miRNA–mRNA expression datasets

To evaluate statistical approaches for miRNA–mRNA integration, we simulated paired miRNA and mRNA expression datasets with controlled levels of anti-correlation between miRNAs and their target genes. Data were generated using a custom R script. Each simulation mimicked a two-group experiment (e.g. case vs. control) comprising 100 samples (50 per group), 2,500 miRNAs, and 20,000 genes.

For each simulated dataset, baseline log-mean expression levels for miRNAs and genes were drawn from normal distributions ($\mu_{miRNAs} = 2$; $\mu_{genes} = 3$) with standard deviations of 0.4. Individual sample expression values were then simulated from log-normal distributions with sample-specific noise ($\sigma_{samp} = 0.2$). A subset of 50 miRNAs was randomly designated as differentially expressed (DE), with random up- or down-regulation between groups and log2-fold changes drawn from a normal distribution ($\mu = 1$; $\sigma = 0.4$). Approximately 30% of these DE-miRNAs were defined as active regulators that exerted expression changes on their target genes.

Each miRNA was assigned a random number of target genes sampled from a negative binomial distribution ($\mu = 300$ targets). To mimic non-uniform probabilities of being targeted, genes were assigned varying susceptibilities to miRNA regulation using a beta distribution ($\alpha = 2$; $\beta = 100$), from which target selection probabilities were derived. For each active miRNA, a proportion of its targets (30% on average) were affected by miRNA-mediated repression.

S5.2 Modelling miRNA–mRNA regulation

To simulate miRNA-mediated repression, gene expression values were perturbed according to a sigmoidal Hill-type model parameterized by random kinetic constants and repression efficiencies. This model is well suited to describe post-transcriptional miRNA-mediated regulation, as it accounts for key features of this process, including multiple binding sites on the same target, cooperativity, saturation effects, and threshold-like behavior [33, 34]. The quantitative kinetic model was originally proposed by Khanin and Vinciotti [35] and was later shown to be among the most efficient approaches for computational modeling of RNA interference [36].

For each regulated gene g, the total repression factor s is defined as the sum of the individual contributions from all regulating miRNAs, as given by Equation 1:

$$s = \sum_{i=1}^{n} E \cdot \frac{m_i^h}{K^h + m_i^h}, \tag{1}$$

where n is the total number of miRNAs regulating gene g, and E is the maximal repression efficiency, ranging from 20% to 50%, consistent with the observation that miRNA regulation tends to be broad but modest in effect [33]. m_i denotes the expression of the ith miRNA, h is

the Hill coefficient (uniformly sampled from $[2, 4]$), and K is the concentration at which half of the maximal mRNA repression is achieved, sampled in our simulations from $[4, 12]$.

The total perturbation for gene g was finally computed as $1-\frac{s}{1+s}$. The resulting perturbation matrix was applied to the log2-transformed gene expression matrix (with scaling factor $\alpha = 4$), and the perturbed matrix was back-transformed to linear space.

Furthermore, to increase analytical complexity and introduce noise, an additional set of 1,000 genes was randomly selected as independent DEGs (unrelated to miRNA regulation), with log2-fold changes drawn from the same distribution as for miRNAs. Lastly, independent log-normal noise ($\sigma = 0.1$) was added to both miRNA and gene matrices to emulate technical variability.

Each simulation returned (i) the miRNA and gene expression matrices, (ii) group labels, (iii) the complete miRNA–target mapping, and (iv) the set of true active miRNA–gene pairs (ground truth).

S5.3 Differential expression analysis

For each dataset, differential expression analysis was performed independently on miRNAs and genes using the limma package. Log2-transformed expression values were fitted to a linear model with group as the explanatory variable (Equation 2):

$$Expression \sim \beta_0 + \beta_1 \times Group. \tag{2}$$

Empirical Bayes moderation was applied using the `eBayes` function, and adjusted p-values were computed using the Benjamini–Hochberg (BH) method. Features with adjusted p-values < 0.05 were considered differentially expressed. DE miRNAs and genes were then categorized as up- or down-regulated based on the sign of their log-fold change.

S5.4 Correlation-based integration (paired analyses)

To assess performance on detecting individual anti-correlated miRNA–mRNA pairs, we restricted the analysis to all predicted miRNA–target pairs in which the miRNA and target were both differentially expressed in opposite directions. Each candidate pair was labelled as true if it belonged to the known set of simulated active miRNA–target pairs, and false otherwise. Four correlation-based tests were compared: Pearson's correlation, Spearman's correlation, partial Pearson's correlation, and partial Spearman's correlation. For simple correlations, pairwise correlation coefficients and p-values were computed using `cor.test` in R. For partial correlations, group membership was used as a conditioning variable. BH-adjusted p-values were calculated, and pairs with FDR < 0.05 were considered significant.

For each correlation method, we computed precision, recall, specificity, sensitivity, accuracy, FDR, and area under the ROC (AUC) and precision–recall (AUPRC) curves. Performance metrics were estimated from confusion matrices using the `caret` package [37]. The correlation coefficient was used as the metric for computing AUC and AUPRC.

S5.5 Categorical (unpaired) integration tests

A separate set of statistical approaches was evaluated to identify influential miRNAs — i.e., miRNAs whose target genes collectively exhibited significant differential expression in the opposite direction. For each simulation, up-regulated miRNAs were tested against down-regulated genes and vice versa. Five categorical enrichment methods were tested: Fisher's exact test, Fisher's exact test with Lancaster's mid-P adjustment, Boschloo's exact test, the Fry test

(rotation gene-set testing, implemented in limma), and the CAMERA test (inter-gene correlation–adjusted gene-set test). For the first three methods, 2×2 contingency tables were constructed for each miRNA by comparing the overlap between its target genes and the list of DEGs with the remaining background genes. For Fisher's and Boschloo's tests, one-sided p-values were computed under the hypothesis that miRNA activation leads to repression of its targets. For the fry and CAMERA methods, log2-transformed gene expression values were analyzed using model matrices that included group membership as a covariate.

Significance was determined using FDR < 0.05. For evaluation, miRNAs were labelled active if they belonged to the set of truly regulating miRNAs in the simulation. As with correlation methods, classification metrics (precision, recall, specificity, sensitivity, accuracy, FDR, AUC, and AUPRC) were computed for each categorical test. For computing curves, $-\log_{10} p$ was used for each method.

S5.6 Benchmark procedure

The entire simulation and testing pipeline was repeated 500 times to ensure stable performance estimates. Parallel computation was implemented using the `BiocParallel` framework with 28 cores. For each iteration, the following steps were performed:

1. Simulate a paired miRNA–mRNA experiment.
2. Identify DE-miRNAs and DEGs using limma.
3. Apply correlation-based tests to DE-miRNA/DE-gene pairs with opposite directionality.
4. Apply categorical (unpaired) tests to identify globally influential miRNAs.
5. Compute performance metrics and store the results.

Performance metrics were combined across all iterations, and summary statistics (mean $\pm$ standard deviation across simulations) were reported.

S5.7 Comparison of F1 scores across varying sample sizes

To assess how the performance of correlation-based methods varies with sample size, we repeated the benchmarking procedure described in Section S5.6 using 10, 15, 20, 25, and 30 samples per condition. For each sample size, 100 simulated datasets were generated, and the F1 score of each correlation-based method was recorded.

References

[1] Matthew E. Ritchie et al. "limma powers differential expression analyses for RNA-sequencing and microarray studies". In: *Nucleic Acids Research* 43.7 (Apr. 2015), e47. ISSN: 0305-1048. DOI: `10.1093/nar/gkv007`. URL: `https://doi.org/10.1093/nar/gkv007` (visited on 09/15/2023).

[2] Mark D. Robinson, Davis J. McCarthy, and Gordon K. Smyth. "edgeR: a Bioconductor package for differential expression analysis of digital gene expression data". In: *Bioinformatics* 26.1 (Jan. 2010), pp. 139–140. ISSN: 1367-4803. DOI: `10.1093/bioinformatics/btp616`. URL: `https://doi.org/10.1093/bioinformatics/btp616` (visited on 09/15/2023).

[3] Michael I. Love, Wolfgang Huber, and Simon Anders. “Moderated estimation of fold change and dispersion for RNA-seq data with DESeq2”. In: *Genome Biology* 15.12 (Dec. 2014), p. 550. ISSN: 1474-760X. DOI: 10.1186/s13059-014-0550-8. URL: https://doi.org/10.1186/s13059-014-0550-8 (visited on 09/15/2023).

[4] Charity W. Law et al. “voom: precision weights unlock linear model analysis tools for RNA-seq read counts”. In: *Genome Biology* 15.2 (Feb. 2014), R29. ISSN: 1474-760X. DOI: 10.1186/gb-2014-15-2-r29. URL: https://doi.org/10.1186/gb-2014-15-2-r29 (visited on 09/15/2023).

[5] Elizabeth I. Boyle et al. “GO::TermFinder—open source software for accessing Gene Ontology information and finding significantly enriched Gene Ontology terms associated with a list of genes”. In: *Bioinformatics* 20.18 (Dec. 2004), pp. 3710–3715. ISSN: 1367-4803. DOI: 10.1093/bioinformatics/bth456. URL: https://doi.org/10.1093/bioinformatics/bth456 (visited on 09/15/2023).

[6] Aravind Subramanian et al. “Gene set enrichment analysis: A knowledge-based approach for interpreting genome-wide expression profiles”. In: *Proceedings of the National Academy of Sciences* 102.43 (Oct. 2005). Publisher: Proceedings of the National Academy of Sciences, pp. 15545–15550. DOI: 10.1073/pnas.0506580102. URL: https://www.pnas.org/doi/full/10.1073/pnas.0506580102 (visited on 09/15/2023).

[7] Di Wu and Gordon K. Smyth. “Camera: a competitive gene set test accounting for inter-gene correlation”. In: *Nucleic Acids Research* 40.17 (Sept. 2012), e133. ISSN: 0305-1048. DOI: 10.1093/nar/gks461. URL: https://doi.org/10.1093/nar/gks461 (visited on 09/15/2023).

[8] Guini Hong et al. “Separate enrichment analysis of pathways for up- and downregulated genes”. In: *Journal of The Royal Society Interface* 11.92 (Mar. 2014). Publisher: Royal Society, p. 20130950. DOI: 10.1098/rsif.2013.0950. URL: https://royalsocietypublishing.org/doi/10.1098/rsif.2013.0950 (visited on 09/15/2023).

[9] The Gene Ontology Consortium et al. “The Gene Ontology knowledgebase in 2023”. In: *Genetics* 224.1 (May 2023), iyad031. ISSN: 1943-2631. DOI: 10.1093/genetics/iyad031. URL: https://doi.org/10.1093/genetics/iyad031 (visited on 09/15/2023).

[10] Minoru Kanehisa et al. “KEGG: new perspectives on genomes, pathways, diseases and drugs”. In: *Nucleic Acids Research* 45.D1 (Jan. 2017), pp. D353–D361. ISSN: 0305-1048. DOI: 10.1093/nar/gkw1092. URL: https://doi.org/10.1093/nar/gkw1092 (visited on 01/25/2023).

[11] Marvin Martens et al. “WikiPathways: connecting communities”. In: *Nucleic Acids Research* 49.D1 (Jan. 2021), pp. D613–D621. ISSN: 0305-1048. DOI: 10.1093/nar/gkaa1024. URL: https://doi.org/10.1093/nar/gkaa1024 (visited on 09/15/2023).

[12] Marc Gillespie et al. “The reactome pathway knowledgebase 2022”. In: *Nucleic Acids Research* 50.D1 (Jan. 2022), pp. D687–D692. ISSN: 0305-1048. DOI: 10.1093/nar/gkab1028. URL: https://doi.org/10.1093/nar/gkab1028 (visited on 09/15/2023).

[13] Ramiro Magno and Ana-Teresa Maia. “gwasrapidd: an R package to query, download and wrangle GWAS catalog data”. In: *Bioinformatics* 36.2 (Jan. 2020), pp. 649–650. ISSN: 1367-4803. DOI: 10.1093/bioinformatics/btz605. URL: https://doi.org/10.1093/bioinformatics/btz605 (visited on 09/15/2023).

[14] Elliot Sollis et al. “The NHGRI-EBI GWAS Catalog: knowledgebase and deposition resource”. In: *Nucleic Acids Research* 51.D1 (Jan. 2023), pp. D977–D985. ISSN: 0305-1048. DOI: 10.1093/nar/gkac1010. URL: https://doi.org/10.1093/nar/gkac1010 (visited on 09/15/2023).

[15] Fergal J Martin et al. “Ensembl 2023”. In: *Nucleic Acids Research* 51.D1 (Jan. 2023), pp. D933–D941. ISSN: 0305-1048. DOI: 10.1093/nar/gkac958. URL: https://doi.org/10.1093/nar/gkac958 (visited on 09/15/2023).

[16] Anne-Christin Hauschild et al. “MirDIP 5.2: tissue context annotation and novel microRNA curation”. In: *Nucleic Acids Research* 51.D1 (Jan. 2023), pp. D217–D225. ISSN: 0305-1048. DOI: 10.1093/nar/gkac1070. URL: https://doi.org/10.1093/nar/gkac1070 (visited on 09/15/2023).

[17] Tomas Tokar et al. “mirDIP 4.1—integrative database of human microRNA target predictions”. In: *Nucleic Acids Research* 46.D1 (Jan. 2018), pp. D360–D370. ISSN: 0305-1048. DOI: 10.1093/nar/gkx1144. URL: https://doi.org/10.1093/nar/gkx1144 (visited on 09/15/2023).

[18] Shidong Cui et al. “miRTarBase 2025: updates to the collection of experimentally validated microRNA–target interactions”. In: *Nucleic Acids Research* 53.D1 (Jan. 2025), pp. D147–D156. ISSN: 1362-4962. DOI: 10.1093/nar/gkae1072. URL: https://doi.org/10.1093/nar/gkae1072 (visited on 04/10/2025).

[19] Dan A. Biddle and Scott B. Morris. “Using Lancaster’s mid-P correction to the Fisher’s exact test for adverse impact analyses”. In: *Journal of Applied Psychology* 96.5 (2011). Place: US Publisher: American Psychological Association, pp. 956–965. ISSN: 1939-1854. DOI: 10.1037/a0024223.

[20] R. D. Boschloo. “Raised conditional level of significance for the 2 × 2-table when testing the equality of two probabilities”. en. In: *Statistica Neerlandica* 24.1 (1970). _eprint: https://onlinelibrary.wiley.com/doi/pdf/10.1111/j.1467-9574.1970.tb00104.x, pp. 1–9. ISSN: 1467-9574. DOI: 10.1111/j.1467-9574.1970.tb00104.x. URL: https://onlinelibrary.wiley.com/doi/abs/10.1111/j.1467-9574.1970.tb00104.x (visited on 10/17/2023).

[21] Di Wu et al. “ROAST: rotation gene set tests for complex microarray experiments”. In: *Bioinformatics* 26.17 (Sept. 2010), pp. 2176–2182. ISSN: 1367-4803. DOI: 10.1093/bioinformatics/btq401. URL: https://doi.org/10.1093/bioinformatics/btq401 (visited on 09/15/2023).

[22] Emily Clough and Tanya Barrett. “The Gene Expression Omnibus Database”. en. In: *Statistical Genomics: Methods and Protocols*. Ed. by Ewy Mathé and Sean Davis. New York, NY: Springer, 2016, pp. 93–110. ISBN: 978-1-4939-3578-9. DOI: 10.1007/978-1-4939-3578-9_5. URL: https://doi.org/10.1007/978-1-4939-3578-9_5 (visited on 04/10/2025).

[23] R Core Team. *R: A Language and Environment for Statistical Computing*. Vienna, Austria: R Foundation for Statistical Computing, 2021. URL: https://www.R-project.org/.

[24] John D. Storey. “A Direct Approach to False Discovery Rates”. In: *Journal of the Royal Statistical Society Series B: Statistical Methodology* 64.3 (Aug. 2002), pp. 479–498. ISSN: 1369-7412. DOI: 10.1111/1467-9868.00346. URL: https://doi.org/10.1111/1467-9868.00346 (visited on 05/13/2025).

[25] Huiqing Liu et al. “Identifying mRNA targets of microRNA dysregulated in cancer: with application to clear cell Renal Cell Carcinoma”. In: *BMC Systems Biology* 4.1 (Apr. 2010), p. 51. ISSN: 1752-0509. DOI: 10.1186/1752-0509-4-51. URL: https://doi.org/10.1186/1752-0509-4-51 (visited on 04/28/2025).

[26] Ismael Santa-Maria et al. “Dysregulation of microRNA-219 promotes neurodegeneration through post-transcriptional regulation of tau”. en. In: *The Journal of Clinical Investigation* 125.2 (Feb. 2015). Publisher: American Society for Clinical Investigation, pp. 681–686. ISSN: 0021-9738. DOI: 10.1172/JCI78421. URL: https://www.jci.org/articles/view/78421 (visited on 10/11/2023).

[27] Clara Y. B. Low et al. “Isoform-specific upregulation of FynT kinase expression is associated with tauopathy and glial activation in Alzheimer’s disease and Lewy body dementias”. en. In: *Brain Pathology* 31.2 (2021), pp. 253–266. ISSN: 1750-3639. DOI: 10.1111/bpa.12917. URL: https://onlinelibrary.wiley.com/doi/abs/10.1111/bpa.12917 (visited on 10/11/2023).

[28] Rasko Leinonen et al. “The Sequence Read Archive”. In: *Nucleic Acids Research* 39.suppl_1 (Jan. 2011), pp. D19–D21. ISSN: 0305-1048. DOI: 10.1093/nar/gkq1019. URL: https://doi.org/10.1093/nar/gkq1019 (visited on 10/11/2023).

[29] Arun H Patil and Marc K Halushka. “miRge3.0: a comprehensive microRNA and tRF sequencing analysis pipeline”. In: *NAR Genomics and Bioinformatics* 3.3 (Sept. 2021), lqab068. ISSN: 2631-9268. DOI: 10.1093/nargab/lqab068. URL: https://doi.org/10.1093/nargab/lqab068 (visited on 10/11/2023).

[30] Marcel Martin. “Cutadapt removes adapter sequences from high-throughput sequencing reads”. en. In: *EMBnet.journal* 17.1 (May 2011). Number: 1, pp. 10–12. ISSN: 2226-6089. DOI: 10.14806/ej.17.1.200. URL: https://journal.embnet.org/index.php/embnetjournal/article/view/200 (visited on 10/11/2023).

[31] Ben Langmead et al. “Ultrafast and memory-efficient alignment of short DNA sequences to the human genome”. In: *Genome Biology* 10.3 (Mar. 2009), R25. ISSN: 1474-760X. DOI: 10.1186/gb-2009-10-3-r25. URL: https://doi.org/10.1186/gb-2009-10-3-r25 (visited on 10/11/2023).

[32] Benilton S. Carvalho and Rafael A. Irizarry. “A framework for oligonucleotide microarray preprocessing”. In: *Bioinformatics* 26.19 (Oct. 2010), pp. 2363–2367. ISSN: 1367-4803. DOI: 10.1093/bioinformatics/btq431. URL: https://doi.org/10.1093/bioinformatics/btq431 (visited on 09/15/2023).

[33] Shankar Mukherji et al. “MicroRNAs can generate thresholds in target gene expression”. en. In: *Nature Genetics* 43.9 (Sept. 2011). Publisher: Nature Publishing Group, pp. 854–859. ISSN: 1546-1718. DOI: 10.1038/ng.905. URL: https://www.nature.com/articles/ng.905 (visited on 10/29/2025).

[34] Vinay K. Mayya and Thomas F. Duchaine. “On the availability of microRNA-induced silencing complexes, saturation of microRNA-binding sites and stoichiometry”. In: *Nucleic Acids Research* 43.15 (Sept. 2015), pp. 7556–7565. ISSN: 0305-1048. DOI: 10.1093/nar/gkv720. URL: https://doi.org/10.1093/nar/gkv720 (visited on 10/29/2025).

[35] Raya Khanin and Veronica Vinciotti. “Computational Modeling of Post-Transcriptional Gene Regulation by MicroRNAs”. In: *Journal of Computational Biology* 15.3 (Apr. 2008). Publisher: Mary Ann Liebert, Inc., publishers, pp. 305–316. DOI: 10.1089/cmb.2007.0184. URL: https://www.liebertpub.com/doi/10.1089/cmb.2007.0184 (visited on 10/29/2025).

[36] Giulia Cuccato et al. “Modeling RNA interference in mammalian cells”. In: *BMC Systems Biology* 5.1 (Jan. 2011), p. 19. ISSN: 1752-0509. DOI: 10.1186/1752-0509-5-19. URL: https://doi.org/10.1186/1752-0509-5-19 (visited on 10/29/2025).

[37] Max Kuhn. “Building Predictive Models in R Using the caret Package”. en. In: *Journal of Statistical Software* 28 (Nov. 2008), pp. 1–26. ISSN: 1548-7660. DOI: 10.18637/jss.v028.i05. URL: https://doi.org/10.18637/jss.v028.i05 (visited on 10/28/2025).
