# Supplementary material for: MIRit: an integrative R framework for the identification of impaired miRNA–mRNA regulatory networks in complex diseases
Source: Bioinform Adv. 2026 Feb 13;6(1):vbag042. doi: 10.1093/bioadv/vbag042 (PMC12961272; doi:10.1093/bioadv/vbag042)
Supplement: vbag042_Supplementary_Data [file vbag042_supplementary_data.zip › MIRit_supplementary_figures.pdf]

MIRit: an integrative R framework for the identification of impaired miRNA-mRNA regulatory networks in complex diseases

Jacopo Ronchi and Maria Foti

Supplementary Figures

Figure S1 – The MIRit workflow 1
Figure S2 – FDR of integrative tests and F1 score at varying sample sizes 2
Figure S3 – The functional impact of miRNA dysregulation in ccRCC 3

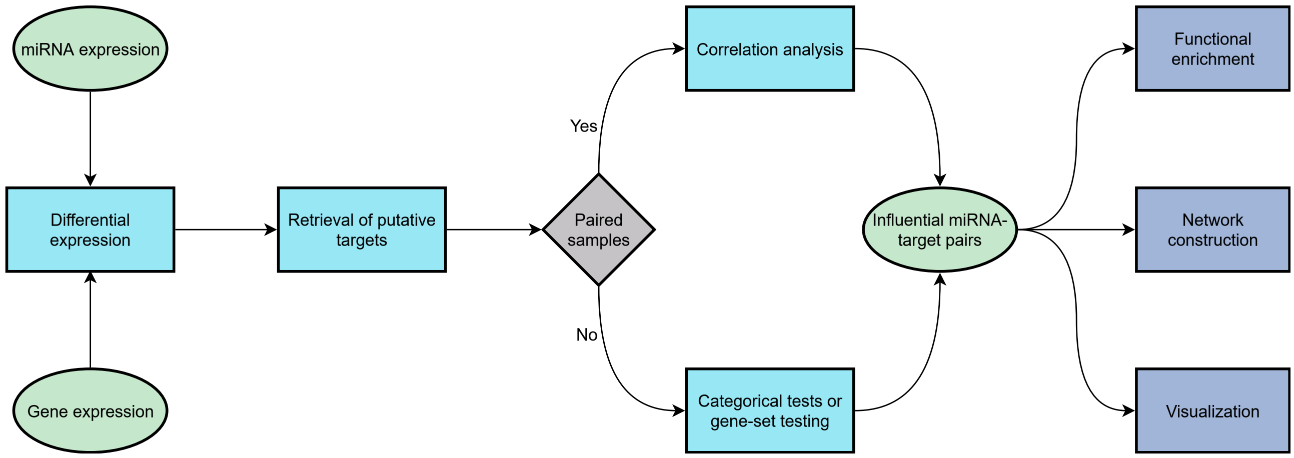

Figure S1: The MIRit workflow. This diagram illustrates the main steps of the MIRit framework. First, differential expression analysis is performed independently for miRNAs and genes, for both microarray and sequencing data. Next, putative miRNA targets are identified by combining predicted interactions from mirDIP and experimentally validated interactions from miRTarBase. miRNA and mRNA expression data are then integrated to identify miRNAs that effectively influence target expression. For paired datasets, integration is based on correlation analysis, whereas for unpaired datasets it relies on categorical tests or rotation gene set testing. Finally, the resulting regulatory networks can be reconstructed, visualized, and functionally enriched.

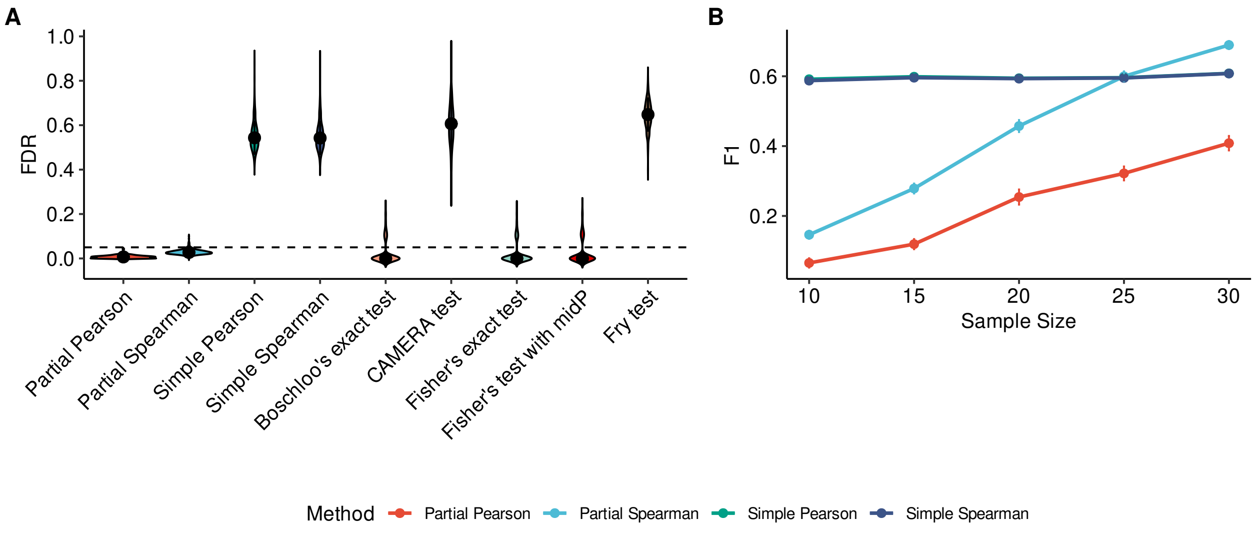

Figure S2: FDR of integrative tests and F1 score at varying sample sizes. **A** shows the FDR of paired and unpaired approaches across 500 simulated datasets. **B** shows the F1 scores of correlation-based approaches at increasing sample sizes.

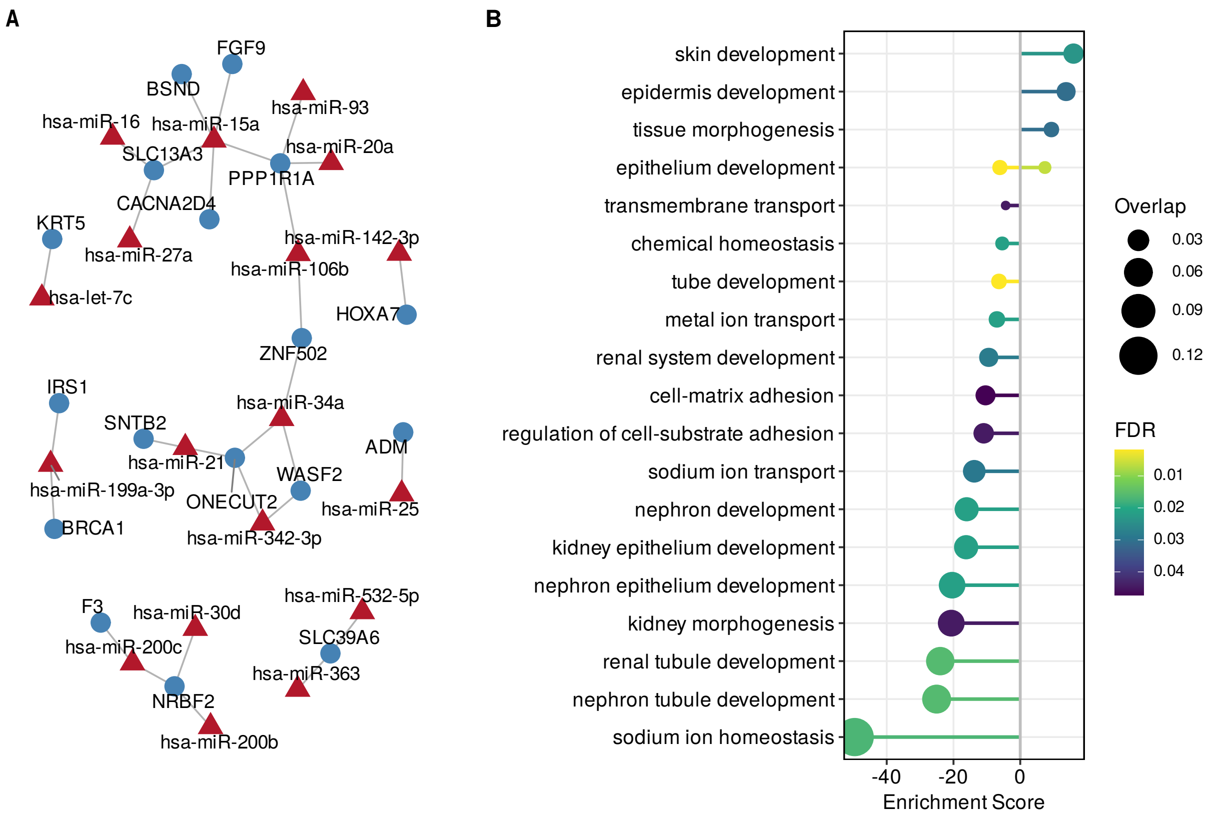

Figure S3: The functional impact of miRNA dysregulation in ccRCC. **A** shows a miRNA-mRNA network with the anti-correlated miRNA-target pairs having a Spearman's correlation coefficient lower than -0.7. In contrast, **B** shows the GO biological processes enriched among the anti-correlated target genes. The Enrichment Score is defined as the fold enrichment of a category, multiplied by +1 if the category is enriched in upregulated genes or by -1 if it is enriched in downregulated genes.
